# Public health implications of vaping in the USA: the smoking and vaping simulation model

**DOI:** 10.1186/s12963-021-00250-7

**Published:** 2021-04-17

**Authors:** David T. Levy, Jamie Tam, Luz María Sanchez-Romero, Yameng Li, Zhe Yuan, Jihyoun Jeon, Rafael Meza

**Affiliations:** 1 Lombardi Comprehensive Cancer Center, Georgetown University, 3300 Whitehaven St, NW, Suite 4100, Washington, DC, 20007 USA; 2 School of Public Health, Yale University, New Haven, CT USA; 3 Department of Epidemiology, University of Michigan, Ann Arbor, MI USA

**Keywords:** NVPs, Vaping, Smoking, Simulation model, Public health

## Abstract

**Background:**

Nicotine vaping products (NVPs) are increasingly popular worldwide. They may provide public health benefits if used as a substitute for smoking, but may create public health harms if used as a gateway to smoking or to discourage smoking cessation. This paper presents the Smoking and Vaping Model (SAVM), a user-friendly model which estimates the public health implications of NVPs in the USA.

**Methods:**

SAVM adopts a cohort approach. We derive public health implications by comparing smoking- and NVP-attributable deaths and life-years lost under a No-NVP and an NVP Scenario. The No-NVP Scenario projects current, former, and never smoking rates via smoking initiation and cessation rates, with their respective mortality rates. The NVP Scenario allows for smoking- and NVP-specific mortality rates, switching from cigarette to NVP use, separate NVP and smoking initiation rates, and separate NVP and smoking cessation rates. After validating the model against recent US survey data, we present the base model with extensive sensitivity analyses.

**Results:**

The SAVM projects that under current patterns of US NVP use and substitution, NVP use will translate into 1.8 million premature smoking- and vaping-attributable deaths avoided and 38.9 million life-years gained between 2013 and 2060. When the NVP relative risk is set to 5%, the results are sensitive to the level of switching and smoking cessation rates and to a lesser extent smoking initiation rates. When the NVP relative risk is raised to 40%, the public health gains in terms of averted deaths and LYL are reduced by 42% in the base case, and the results become much more sensitive to variations in the base case parameters.

**Discussion:**

Policymakers, researchers, and other public health stakeholders can apply the SAVM to estimate the potential public health impact of NVPs in their country or region using their own data sources. In developing new simulation models involving NVPs, it will be important to conduct extensive sensitivity analysis and continually update and validate with new data.

**Conclusion:**

The SAVM indicates the potential benefits of NVP use. However, given the uncertainty surrounding model parameters, extensive sensitivity analysis becomes particularly important.

**Supplementary Information:**

The online version contains supplementary material available at 10.1186/s12963-021-00250-7.

## Background

Smoking prevalence has markedly declined in the USA over the past 50 years [44, 92]. During that time, cigarette taxes were substantially increased, and smoke-free air policies, media campaigns, youth access policies and smoking cessation treatments were implemented [92]. These policies have been shown to be effective [54], and can explain much of the reduction in smoking prevalence [44, 50, 92, 101, 102]. Despite this progress, the harms from cigarette smoking remain unacceptably high [92]. More than 480,000 Americans die each year due to smoking, and two-thirds of long-term smokers die prematurely of a smoking-attributable disease [11, 48, 91]. To drastically reduce the harms of smoking on population health, innovative approaches are needed.

The rising use of nicotine vaping products (NVPs), also known as e-cigarettes or electronic nicotine delivery systems (ENDS), has been a source of both promise and controversy [51, 52]. NVPs, especially later generation models, have been shown to more efficiently deliver nicotine [24, 28, 33, 99, 106] and have sensorimotor effects and throat irritation similar to smoking [26, 34, 38, 98], thus potentially serving as a substitute for cigarettes. NVPs are increasingly being used by smokers to quit [10, 30, 32], and recent studies indicate their effectiveness [9, 39, 55, 107]. If smokers switch entirely to NVPs, the health benefits could be substantial since NVPs deliver only a fraction of the toxicants delivered by smoking [35, 36, 41, 77, 80, 82, 83]. However, there is also concern about increased use of these products by youth [20] and their potential to increase youth cigarette smoking [84, 104], inhibit smoking cessation [70, 73], and promote relapse [5, 22].

The US Food and Drug Administration has the authority to regulate NVPs [29] and must now consider “the risks and benefits of the tobacco product to the population as a whole” [93]. In their review of NVPs, the agency evaluates the toxicity of the product and its effect on the uptake and cessation of existing tobacco products from a public health perspective. The impact of NVP use (“vaping’) on population health will depend on its use in relation to cigarette use (“smoking”) [51, 52]. As a reduced risk alternative, vaping would improve public health when used by those who would have otherwise initiated smoking or by those who would not have otherwise quit smoking. Vaping would worsen public health when used by those who would not have otherwise initiated NVPs or smoking (i.e., as a gateway to smoking) or if used by those who would have otherwise quit smoking. The ability to accurately predict these impacts will depend on the ability to incorporate transitions between NVPs and smoking, but knowledge of the relevant transitions is often lacking.

While many models have been developed that incorporate NVPs [6, 14, 17, 42, 49, 65, 76, 85, 103, 105], these models are often geared towards specific applications, lack the flexibility to be easily applied to other settings, require substantial efforts to understand, require data that is not generally available and have not been validated against real-world data. Because NVP model parameters are subject to considerable uncertainty, it is also important that sensitivity analysis can be easily conducted.

We present the Smoking and Vaping Model (SAVM), which aims to bridge these gaps. This user-friendly model is based on an earlier model [53], but provides greater flexibility by incorporating a wider range of initiation, cessation and switching parameters and can easily be adapted to other countries, states, or local areas. The SAVM differs from previous work in four ways. First, the SAVM adopts a cohort-based approach, thereby incorporating any dependence of NVP use patterns on the availability of NVPs and current policies in effect at a particular age and year (e.g., adolescents who initiated e-cigarette use prior to 2013 had less exposure to NVPs than adolescents who initiated after 2013). Second, the model has a simplified structure and is available in Excel, making it transparent and more easily adapted and applied. Third, the model is validated against recent national survey data. Finally, the model is developed to conduct comprehensive sensitivity analyses of the NVP parameters. This study presents the model and demonstrates its application to the USA.

## Methods

The public health impact of NVP use among smokers and non-smokers is estimated by comparing two scenarios: a) the *No-NVP Scenario* which projects future cigarette use and associated mortality outcomes for each birth cohort in the absence of NVPs, and b) the *NVP Scenario* which incorporates NVP use patterns into each cohort’s cigarette use trajectory. To simplify the model and ensure that health outcomes reflect regular (i.e., stable) use patterns over time [48], we focus on the regular (rather than experimental) use of NVPs and cigarettes and the transitions between those uses.

### The No-NVP Scenario

**Fig. 1:**
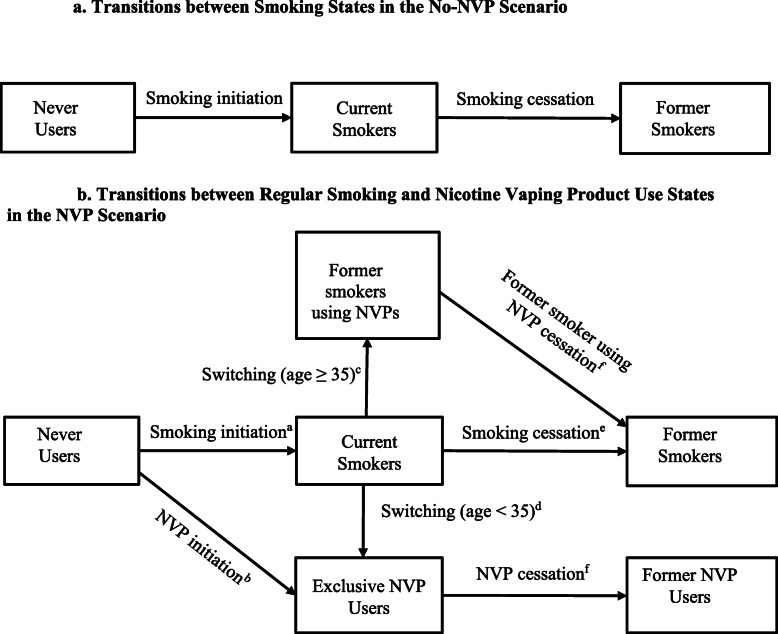
**a** Transitions between smoking states in the No-NVP Scenario. **b** Transitions between regular smoking and nicotine vaping product use states in the NVP Scenario. NVP, nicotine vaping product. ^a^Smoking initiation includes initiation into regular cigarette use net of any experimental NVP use and includes those who become regular dual users who smoke and regularly use NVPs. ^b^NVP initiation includes initiation into regular NVP use net of any experimental cigarette use. ^c^Switching after age 35 includes those regular smokers who quit smoking and switch to regular NVP and are considered before former smokers using NVPs. ^d^Switching before age 35 includes those regular smokers who quit smoking and switch to regular NVP use, and considered exclusive NVP users due to the reduced risks of quitting smoking before age 35. ^e^Smoking cessation includes cessation from regular cigarette use and dual users who quit both cigarette and NVP use, but may include those who quit smoking and temporarily use NVP. ^f^Former smoker using NVPs cessation includes those former smokers who quit NVPs and remain former smokers. NVP cessation includes those exclusive NVP users who quit NVP use

The SAVM begins with separate cohorts of males and females by individual age. Within each cohort, the population evolves with age. The No-NVP Scenario projects the prevalence of current and former smokers over time using age- and gender-specific initiation and cessation rates for each cohort that were previously developed using an age-period-cohort statistical smoking model [43, 45, 47, 88]. The initiation and cessation rates are projected forward based on data from the US National Health Interview Survey (NHIS) through the year 2013, before NVPs were in more widespread use [58] and thus reflects smoking patterns in the absence of NVPs. The transitions are shown in Fig. 1a.

Individuals are born as never users, which in the context of the No-NVP model is just never smokers. For a given cohort, never users at age a and year t includes surviving never users in the previous year (age a-1, year t-1) who did not initiate smoking, where surviving never users are modeled as never users at age a-1 in year t-1 multiplied by (1—mortality rate of never users_a-1,t-1_)*.* Smoking initiation among never users can occur through age 40 and reflects the transition to becoming an established current smoker, defined as having smoked at least 100 cigarettes during one’s lifetime and currently smoking every day or some days. Smokers at age a and year t include surviving never users at age a-1 and year t-1 who initiated smoking and surviving smokers (using current smoker mortality rates) who did not quit since the previous year (age a-1, year t-1). Smoking cessation among current smokers reflects the permanent transition to becoming a former smoker and requires that the smoker quit at least 2 years; this avoids the need to model relapse and reflects the underlying inputs used for this and other tobacco models [44, 45, 88]. Former smokers include surviving former smokers (based on age- and former smoker-specific mortality rates) and surviving smokers who quit smoking for at least 2 years.

### The NVP Scenario

Starting from the same initial current, former, and never smoking prevalence as in the No-NVP Scenario, the NVP Scenario is expanded to include current and former NVP use. The transitions are shown in Fig. 1b.

Because SAVM focuses on measures of regular use, the NVP Scenario does not explicitly model individual youth transitions directly from short-term vaping to regular smoking. Any such transitions are however incorporated indirectly through a smoking initiation multiplier reflecting the net effect of vaping on smoking initiation rates. Similarly, the NVP Scenario does not explicitly model short-term NVP use leading to quitting both cigarettes and NVPs, but is indirectly incorporated through a smoking cessation multiplier reflecting the net effect of vaping on smoking cessation rates. In addition, the NVP Scenario does not distinguish dual cigarette and NVP users from exclusive smokers, because dual users have generally been found to either remain dual users or soon transition to either exclusive cigarette or exclusive NVP use [4, 8, 18, 78, 86, 89] and the health risks of dual users appear to be similar to those of exclusive smokers [36, 82, 83]. However, transitions from dual use to exclusive NVP use or neither cigarette nor NVP use are reflected in the switching and smoking cessation parameters.

Never users include surviving never users from the previous age and year who do not initiate into regular smoking or NVP use. The model directly relates the NVP Scenario to the No-NVP Scenario through separate linear multipliers applied to the smoking initiation rate in the No-NVP Scenario. Using a linear multiplier assures that NVP and smoking initiation follow the same age patterns for smoking initiation as in the No-NVP case, e.g., smoking initiation mostly occurs before age 21 and is minimal after age 30. NVP initiation is similarly tied to smoking initiation in the No-NVP Scenario, whereby a multiplier value less than (or greater than) 100% implies that NVP initiation rates are less than (or greater than) smoking initiation rates in the No-NVP Scenario.

In the NVP Scenario, smokers become former smokers in one of two ways: (1) they may switch to regular exclusive NVP use, or (2) they may quit both regular smoking and regular NVP use (e.g., dual users would quit both) and thereby become former smokers. In the latter case, the smoker may temporarily use NVPs but quit both smoking and vaping. The overall reduction in smokers in the NVP Scenario is the reduction in smokers from switching and complete cessation, and this sum may be less (e.g., harm reduction) or more (harm increasing) than the number of smokers in the No-NVP Scenario. Smokers age a in year t include surviving smokers at age a-1 and year t-1 who did not switch to exclusive vaping and who did not quit smoking, and surviving never users at age a-1 and year t-1 who initiated smoking.

Similar to the initiation process, smoking and vaping cessation are modeled as separate linear multipliers of smoking cessation in the No-NVP Scenario, so that smoking cessation follows the same age pattern in the No-NVP Scenario and tends to increase with age. Since studies to date do not indicate different age patterns, the smoking and vaping initiation and cessation multipliers are simply modeled as independent of age. In addition, these multipliers are assumed to remain constant over time, so that smoking and vaping initiation and cessation in the NVP Scenario follow the same temporal patterns as in the No-NVP Scenario.

Former smokers who either switch to or quit smoking before age 35 are distinguished from those who quit or switch at age 35 or above. Those who quit smoking (by switching to NVPs or cessation) before age 35 are classified the same as never smokers who vape rather than as former smokers, because mortality risks of smoking are minimal when quitting before age 35 [48]. Those who quit at age 35 or above maintain the former smoker status and its resulting mortality risks. Thus former smokers include surviving former smokers, surviving smokers (age > 35) who quit smoking and do not vape, surviving smokers who switched to vaping (age > 35), and surviving former smokers who at one point regularly vaped but quit vaping.

Exclusive NVP users include surviving never users who initiate vaping, surviving vapers who do not quit, and those switching to vaping from surviving current smokers before age 35. Similar to smokers, those who quit smoking before age 35 are treated the same as never smokers who vape rather than former smokers. Since former smokers using NVPs may quit vaping, former smokers using NVPs includes surviving former smokers using NVPs who do not quit vaping and smokers who switch to vaping (after age 35).

A detailed discussion of the parameters and equations used in both scenarios can be found in Supplement 1.

### Public health outcomes

The SAVM considers two public health outcomes: (1) Smoking- and vaping-attributable deaths and (2) smoking- and vaping-attributable life-years lost (LYLs). Both are based on the excess risks of smoking or vaping and the number of current and former smokers and vapers.

In the No-NVP Scenario, smoking-attributable deaths (SADs) by age and gender for current smokers are calculated by applying the excess risks of current smokers relative to never smokers (current smoker mortality rate_a,t_ − never smoker mortality rate_a,t_) to the smoking population and for former smokers are calculated by applying the excess risks of former smokers relative to never smokers (former smoker mortality rate_a,t_ − never smoker mortality rate_a.t_) to the former smoker population. SADs for current and former smokers are summed over all ages in a particular year to obtain total SADs in that year. LYLs are estimated as the number of premature deaths multiplied by the remaining life expectancy of a never smoker at age a in year t. The sum over all ages in a particular year obtains the LYLs in that year.

In the NVP Scenario, current and former smoker SADs are calculated in the same manner in the No-NVP Scenario as above except using their respective current and former smoker population sizes from the NVP scenario. For current and former exclusive NVP users, attributable deaths are a product of the number of NVP users and the excess risks of smoking adjusted by NVP relative risk, denoted by Risk_NVP_, i.e., Risk_NVP_*(current smoker mortality rate_a,t_ − never smoker mortality rate_a,t_) for current users and Risk_NVP_*(former smoker mortality rate_a,t_ − never smoker mortality rate_a,t_) for former users. As a special case in the NVP Scenario, the mortality rate of NVP users who previously smoked is determined by the mortality rate of former smokers plus the portion of excess NVP risk of current relative to former smokers. Smoking and vaping-attributable deaths (SVADs) for former smokers who currently use NVPs is measured as a product of the number of former smokers who currently use NVPs and the excess mortality risk of these users to never smokers, calculated as (former smoker using NVPs mortality rate_a,t_ − never smoker mortality rate_a,t_). Total attributable deaths are calculated by summing SVADs over all ages in each year. LYLs in the NVP Scenario at each age are calculated as the product of SVADs and life-years remaining of never smokers. Summing over all ages in a particular year obtains the total LYLs in that year.

The public health impact of NVP use each year is evaluated as the difference in attributable deaths between the No-NVP and NVP Scenarios, and similarly for LYLs.

#### Data and parameter specification

In SAVM, the analysis of current, former and never smoking and vaping is in terms of their prevalence. These prevalence rates are translated into population numbers using actual and projected US population size estimates [12]. While the model itself does not explicitly incorporate projected births, mortality, and immigration, the CDC projections incorporate projected births, mortality, and immigration.

A detailed description of model parameters is provided in Table 1.

**Table 1:** Data and initial parameters for the US Smoking and Vaping Model

Input parameters	Description	Data source or estimate
Population	Population by age, gender, and year (2013–2060)	US population projections [12, 13]
Mortality rates	Mortality rates by age, gender, and year for never, current, and former smokers (2013–2060)	CISNET Lung Group, ([43], Holford, [50, 47, 88]) available on the CISNET website [16].
Expected life years	Expected life years remaining of never smokers by age, gender, and year (2013–2060)	CISNET Lung Group, ([43, 45, 47, 88]) available on the CISNET website [16].
Smoking prevalence	Current and former smoking prevalence by age and gender for initial year	CISNET Lung Group, ([43], Holford, [50, 47, 88]) available on the CISNET website [16].
NVP relative risk multiplier	Excess risk of NVP use measured relative to excess smoking risks (mortality rate of current smokers − mortality rate of never smokers)	NVP mortality risks estimated to be 5% that of smoking excess risk for both genders at all ages, based on multi-criteria decision analysis [75] and independent review [64].
NVP switching rate	Rate of switching from smoking cigarettes to exclusive NVP use	Ranges from 0.6–8.0%, estimated by age group and gender using prospective analysis from PATH data 2013–2018
Smoking initiation multiplier in the NVP Scenario	Ratio of smoking initiation rate in the NVP Scenario to the No-NVP Scenario	75% of No-NVP smoking initiation rate, based on [57].
NVP initiation multiplier in the NVP Scenario	Ratio of NVP initiation rate in the NVP Scenario to the No-NVP Scenario	50% of No-NVP smoking initiation rate, based on recent studies ([20, 40, 68, 69]).
Smoking cessation multiplier in the NVP Scenario	Ratio of smoking cessation rate in the NVP Scenario to the No-NVP Scenario	100% of the No-NVP smoking cessation rate
NVP cessation multiplier in the NVP Scenario	Ratio of NVP cessation rate in the NVP Scenario to the No-NVP Scenario	100% of the No-NVP smoking cessation rate

No-NVP Scenario refers to values in the absence of NVP use. NVP Scenario refers to values with NVP use

*NVP* nicotine vaping product

#### The No-NVP Scenario

The initial level of current, former and never smoking prevalence, the smoking initiation and cessation rates used to project these future prevalence rates, mortality rates by smoking status, and the life expectancy of never smokers used in the No-NVP model were previously developed [43–45, 79] available on the National Cancer Institute: Cancer Intervention and Surveillance Modeling Network (CISNET) website [16]. These measures apply data only through 2013 to incorporate trends prior to the time when NVPs became more widely used.

#### The NVP Scenario

*The NVP Scenario requires six* input parameters: NVP mortality risks, NVP switching rate, smoking initiation, vaping initiation, smoking cessation, and vaping cessation.

The NVP relative risk multiplier*,* Risk_NVP_, represents the relative risk of death associated with current NVP use as a percentage of the excess mortality risk experienced by current or former smokers as defined above. NVP relative mortality risk is designated at 5% of cigarette excess risks based on a multi-criteria decision analysis [75] and a Public Health England review [64]. Since others have suggested higher risks [23, 74, 90, 95], an NVP relative risk multiplier of 40% is also considered.

The NVP switching rate is the annual rate at which current smokers switch from smoking to NVP use, leading to a direct *reduction in smoking prevalence.* Baseline male (female) NVP yearly switching rates are based on weighted data by age group that we generated from the 2013/2014, 2014/2015, 2015/2016, and 2016/2017 Population Assessment of Tobacco and Health (PATH) surveys [94] averaged over years, as described in Supplement 2. These are: 8% (5%) age 10–17; 4.0% (2.5%) for ages 18–24, 2.5% (2.0%) for ages 25–34, 2.5% (1.6%) for age 35–44, 1.3% (1.4%) for ages 45–54, 1.2% (1.4%) for ages 55–64, and 0.6% (1.0%) for ages 65. We also consider switching rates that are 50% lower and 100% higher than the baseline estimates. These rates are initially assumed constant over time (i.e., 0% decay), but we also consider a 10% decay (i.e., annual rate of decline) to reflect the possibility of reduced innovation in NVPs over time and the tendency for those who are most amenable to switching to or quitting NVPs to have already switched, leaving a population less amenable to switching and quitting. However, because innovation in NVP design may increase their substitutability for cigarettes and potentially increase switching, we also consider an annual 5% increase rate for the first five years (2018–2022).

Since the smoking initiation in the NVP Scenario is measured relative to smoking initiation in the No-NVP Scenario, the smoking initiation multiplier is greater than 100% if NVP use increases net smoking initiation beyond the rate at which individuals would have otherwise initiated smoking in the absence of NVPs (i.e., gateway in > gateway out), and is less than 100% if those who would have initiated smoking tend to transition to exclusive NVP use instead of smoking (i.e., gateway out > gateway in). Based on the more rapid downward trend in US youth and young adult smoking as NVP use increased in recent years [57], the age- and year-invariant baseline smoking initiation multiplier is initially set at 75%, i.e., a 25% net decrease in smoking initiation due to vaping. A range of 25 to 125% is also considered.

The NVP initiation rate multiplier reflects the youth and young adult initiation of NVP use relative to smoking initiation rates in the No-NVP Scenario. If less than 100%, NVP initiation rates are lower than smoking initiation rates in the No-NVP Scenario. i.e., fewer individuals become regular NVP users than who would have become smokers in the No-NVP Scenario. Reflecting the increased US youth and young adults regular NVP users in recent years [20, 40, 51, 52, 68, 69], the age- and year-invariant baseline NVP initiation multiplier is initially set at 50%. Sensitivity analysis is conducted over the range of 25 to 75%.

Since smoking cessation in the NVP Scenario is measured relative to smoking cessation in the No-NVP Scenario, the smoking cessation multiplier is greater than 100% if smoking cessation rates are higher in the NVP Scenario than in the No-NVP Scenario, e.g., if the availability of NVPs leads to increased smoking cessation. A parameter less than 100% implies that smokers are less likely to quit smoking in the NVP Scenario compared with the No-NVP Scenario, e.g., NVP use leads to dual use (continued smoking) rather than complete cessation among smokers. The baseline smoking cessation rate multiplier is initially set at 100%, with sensitivity analysis at 50% and 150%.

The NVP cessation multiplier is greater than 100% if NVP cessation rates are higher than smoking cessation rates in the No-NVP Scenario, e.g., NVPs are less addictive than cigarettes. The parameter is less than 100% if NVP cessation rates are lower than smoking cessation rates in the No-NVP Scenario. The baseline NVP cessation multiplier is set at 100%, with sensitivity analysis at 50% and 150%.

### Model validation and analysis

The model estimates the NVP effects over time for the prevalence of current and former smokers, current and former NVP users, and former smokers using NVPs, and for smoking-attributable and NVP-attributable deaths and LYLs. The model was first validated over the years 2013 to 2018 by comparing model predictions of current smoking prevalence to current smoking prevalence rates from the NHIS [13]. We focus on relative reductions over the period 2013–2018, because levels of model smoking prevalence in the initial year 2013 differ from those in the NHIS.

We also validated NVP use. Although NVP use was already occurring in 2013, the model itself begins with no NVP use that year and is adjusted to reach the 2018 levels [57]. Consequently, we validated NVP use prevalence against NHIS estimates for 2018, the latest year for which data was available rather than rely on prior trend data. We defined regular NVP users as those who used NVPs at least 10 of the past 30 days to reflect more regular use [3, 58].

Upon validating the model, we consider how NVP use affects smoking prevalence, smoking-attributable and NVP-attributable deaths, and LYLs. We conduct sensitivity analyses for the NVP transition and risk parameters over the plausible ranges specified above focusing on their impact on premature attributable deaths and LYLs.

## Results

### Validation of smoking prevalence

Using the initial (best estimate) input parameter estimates, the SAVM predictions are validated against smoking prevalence from NHIS data for 2013–2018 as shown in Table 2. For ages 18 and above, SAVM projects that male (female) smoking prevalence fell from 21.4% (15.9%) in 2013 to 16.7% (12.6%) in 2018, while NHIS shows a decline from 20.3% (15.4%) in 2013 to 15.8% (12.0%) in 2018. NHIS shows a 22.2% (22.1%) relative reduction between 2013 and 2018 compared with a 22.2% (20.7%) relative reduction in SAVM.

**Table 2:** Smoking prevalence (%), validation of US SAVM against the NHIS, by age and gender, 2013–2018

Age	Source	2013	2014	2015	2016	2017	2018	Relative difference 2013–2018
**US males**								
**18+**	SAVM	21.4	20.4	19.4	18.5	17.5	16.7	− 22.2%
	NHIS	20.3	18.8	16.7	17.5	15.8	15.8	− 22.2%
	95% CI	19.5**–**21.2	17.9**–**19.7	15.9**–**17.6	16.7**–**18.4	15.0**–**16.6	15.0**–**16.6	
**18–24**	SAVM	19.9	18.2	16.6	15.3	14.2	13.3	− 33.0%
	NHIS	21.6	18.6	15.2	14.6	12.1	8.5	− 60.8%
	95% CI	18.6**–**24.6	15.9**–**21.4	12.5**–**18.0	11.9**–**17.2	9.7**–**14.5	6.4**–**10.5	
**25–44**	SAVM	27.3	26.3	25.2	24.0	22.8	21.6	− 21.0%
	NHIS	23.1	22.9	19.7	20.6	19.3	19.1	− 17.2%
	95% CI	21.6**–**24.5	21.3**–**24.4	18.2**–**21.3	19.0**–**22.2	17.7**–**20.8	17.5**–**20.7	
**45–64**	SAVM	20.4	19.5	18.6	17.8	17.1	16.3	− 19.9%
	NHIS	21.6	19.3	17.8	19.4	17.3	18.3	− 15.2%
	95% CI	20.2**–**23.1	17.7**–**20.9	16.5**–**19.2	18.0**–**20.8	15.9**–**18.6	16.9**–**19.7	
**65+**	SAVM	12.2	11.9	11.5	11.0	10.6	10.2	− 16.6%
	NHIS	10.6	9.7	9.8	10.2	9.0	9.9	− 6.3%
	95% CI	9.3**–**12.0	8.4**–**11.0	8.5**–**11.1	8.9**–**11.5	7.9**–**10.2	8.7**–**11.1	
**US females**								
**18+**	SAVM	15.9	15.2	14.5	13.8	13.2	12.6	− 20.7%
	NHIS	15.4	14.9	13.5	13.6	12.1	12.0	− 22.1%
	95% CI	14.7**–**16.1	14.1**–**15.7	12.8**–**14.2	12.9**–**14.2	11.5**–**12.8	11.3**–**12.6	
**18–24**	SAVM	15.0	13.7	12.5	11.5	10.6	9.9	− 33.7%
	NHIS	15.4	14.8	11.1	11.4	8.5	7.3	− 52.6%
	95% CI	13.0**–**17.8	10.6**–**18.9	9.0**–**13.3	9.4**–**13.3	6.5**–**10.5	5.2**–**9.4	
**25–44**	SAVM	20.6	19.9	19.2	18.5	17.7	16.9	− 17.6%
	NHIS	17.0	17.4	15.6	14.7	12.9	14.2	− 16.6%
	95% CI	15.8**–**18.2	16.1**–**18.6	14.4**–**16.9	13.6**–**15.9	11.8**–**14.1	12.9**–**15.5	
**45–64**	SAVM	16.2	15.6	14.9	14.4	13.8	13.3	− 18.1%
	NHIS	18.1	16.8	15.8	16.8	15.3	14.3	− 20.9%
	95% CI	16.8**–**19.3	15.6**–**18.0	14.6**–**17.1	15.6**–**18.0	14.1**–**16.5	13.1**–**15.5	
**65+**	SAVM	8.2	7.8	7.4	7.1	6.8	6.5	− 20.3%
	NHIS	7.6	7.6	7.6	7.6	7.8	7.3	− 4.0%
	95% CI	6.6**–**8.6	6.5**–**8.6	6.5**–**8.6	6.7**–**8.6	6.8**–**8.7	6.4**–**8.2	

Current smokers in the NHIS (National Health Interview Survey) are those who have smoked at least 100 cigarettes during one’s lifetime and currently smoke cigarettes some days or every day

**Fig. 2:**
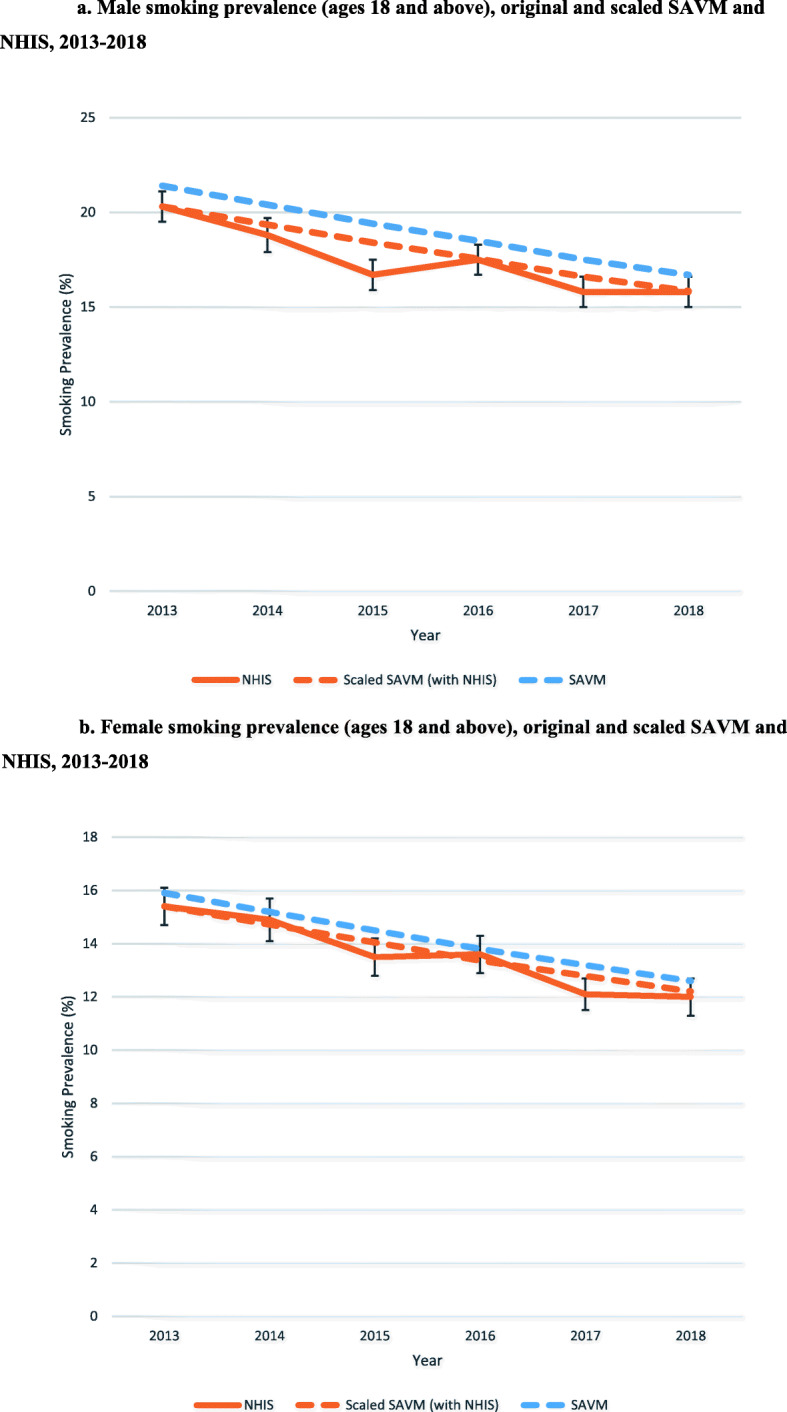
**a** Male smoking prevalence (ages 18 and above), original and scaled SAVM and NHIS estimates, 2013–2018. **b** Female smoking prevalence (ages 18 and above), original and scaled SAVM and NHIS estimates, 2013–2018

The SAVM and NHIS smoking prevalence for males ages 18 and above is shown in Fig. 2a (including NHIS estimates with 95% confidence intervals) with male SAVM prevalence rates scaled by the ratio of survey estimate to the SAVM level in 2013, e.g., NHIS 20.3%/ SAVM 21.4% = 94.9%). Female prevalence rates are shown in Fig. 2b., with female SAVM prevalence scaled by 96.9%.

By age group, SAVM projects that male (female) prevalence for ages 18–24 fell by 33.0% (33.7%) in relative terms between 2013 and 2018 compared with 60.8% (52.6%) from NHIS over the same time period. For ages 25–44, SAVM projects a relative decline of 21.0% (17.6%) compared with 17.2% (16.6%) from NHIS. For ages 45–64, SAVM projects a relative decline of 19.9% (18.1%) compared with 15.2% (20.9%) from NHIS. For ages 65 and above, SAVM projects a relative decline of 16.6% (20.3%) compared with 6.3% (4.0%) from NHIS.

Thus, while predicting smoking prevalence well for the overall adult population, SAVM tends to under-predict relative reductions in smoking prevalence at younger ages and over-predict relative reduction at older ages. While SAVM estimates for 2018 are often outside the NHIS confidence intervals, upon scaling all years by the initial year (2013) from SAVM relative to the initial year in NHIS for comparability, the SAVM 2018 estimates are generally within the NHIS confidence intervals, with the exceptions of the 18–24 age group for males and the 65 and above age group for females.

### Validation of NVP prevalence

The SAVM predictions for exclusive NVP use are validated against the overall NVP prevalence from NHIS for 2018 as shown in Table 3. For ages 18 and above, SAVM projects a 2018 NVP prevalence of 3.0% compared with 3.1% (95% CI: 2.7–3.5%) from NHIS for males, and 1.8% compared with 1.5% (95% CI: 1.3–1.8%) for females. By age group for males, the SAVM projection against NHIS (with confidence intervals) for 2018 are 7.5% vs 6.8% (95% CI:4.8–8.8%) for ages 18–24, 4.2% vs. 4.7% (95% CI: 3.8–5.6%) for ages 25-44, 1.4% vs. 1.5% (95% CI: 1.0–1.9%) for ages 45–64, and 0.5% vs. 0.6% (95% CI: 0.3–0.9%) for ages 65 and above. For females, SAVM projections vs. NHIS estimates are 4.9% vs. 3.1% (95% CI: 1.8–4.5%) for ages 18–24, 2.4% vs. 1.9% (95% CI: 1.4–2.3%) for ages 25–44, 1.0% vs. 1.3% (95% CI: 1.0–1.7%) for ages 45–64, and 0.4% vs. 0.5% (95% CI: 0.3–0.7%) for ages 65 and above. SAVM predictions fell within the 95% confidence intervals estimated from NHIS data for overall and by age groups, except for females ages 18–24 and 25-44.

**Table 3:** Nicotine vaping product prevalence (%), validation for US SAVM against NHIS by age and gender in the year 2018

Ages	18+	18–24	25–44	45–64	65+
**US males**					
SAVM	3.0	7.5	4.2	1.4	0.5
NHIS	3.1	6.8	4.7	1.5	0.6
NHIS 95% CI	2.7**–**3.5	4.8**–**8.8	3.8**–**5.6	1.0**–**1.9	0.3**–**0.9
**US females**					
SAVM	1.8	4.9	2.4	1.0	0.4
NHIS	1.5	3.1	1.9	1.3	0.5
NHIS 95% CI	1.3**–**1.8	1.8**–**4.5	1.4**–**2.3	1.0**–**1.7	0.3**–**0.7

NVP users from NHIS (National Health Interview Survey) are those who use NVPs at least 10 of the past 30 days

*CI*, confidence interval

### Public health impact under the baseline NVP and No-NVP Scenario

Table 4 presents US smoking and NVP use prevalence, deaths, and LYLs for males and females ages 18–99 over the modeling period 2013–2060 for all existing and new birth cohorts. We focus on prevalence estimates for the year 2023 as an indication of short-term projection and for the year 2060 as an example of long-term projections. We first apply the baseline parameters: Risk_NVP_ = 5% that of smoking, with multipliers for smoking initiation = 75%, NVP initiation = 50%, smoking cessation = 100%, NVP cessation = 100%, and with switching rates remaining constant over time.

**Table 4:** Baseline SAVM outcomes under NVP Scenario vs. No-NVP Scenario, all cohorts including new births, US, by gender, in years 2013–2060, ages 18–99

	Year	2013	2018	2023	2060	Cumulative^**a**^
**US males**						
**No-NVP Scenario**^b^	Smokers (%)	21.4%	19.2%	17.4%	12.2%	–
	SADs	280,925	277,980	274,943	219,947	12,555,272
	LYLs	3,668,313	3,551,197	3,389,252	2,275,317	141,511,570
**NVP Scenario**^c^	Smokers (%)	21.4%	16.7%	12.9%	4.5%	–
	NVP users (%)	0.0%	2.1%	3.8%	8.8%	–
	FS-NVP users (%)	0.0%	0.9%	1.5%	1.6%	–
	SVADs	280,925	271,354	261,620	171,734	11,264,274
	LYLs	3,668,313	3,406,256	3,098,328	1,243,989	112,777,500
**Difference**	SADs averted	0	6,626	13,323	48,214	1,290,997
	LYLs averted	0	144,941	290,924	1,031,327	28,734,070
**US females**						
**No-NVP Scenario**	Smokers (%)	15.9%	14.1%	12.7%	8.7%	–
	SADs	110,815	106,875	106,031	86,296	5,024,120
	LYLs	1,405,272	1,369,936	1,318,238	822,514	54,081,266
**NVP Scenario**	Smokers (%)	15.9%	12.6%	10.1%	4.1%	–
	NVP users (%)	0.0%	1.2%	2.2%	5.5%	–
	FS-NVP users (%)	0.0%	0.6%	1.0%	1.0%	–
	SVADs	110,815	103,063	98,981	68,794	4,481,391
	LYLs	1,405,272	1,301,979	1,192,904	485,330	43,949,321
**Difference**	SADs averted	0	3,812	7,050	17,502	542,729
	LYLs averted	0	67,957	125,335	337,185	10,131,945
**Both genders**						
**Difference**	SADs averted	0	10,438	20,373	65,716	1,833,727
	LYLs averted	0	212,898	416,259	1,368,512	38,866,015
	SADs averted (%)	0.0%	2.7%	5.3%	21.5%	10.4%
	LYLs averted (%)	0.0%	4.3%	8.8%	44.2%	19.9%

*NVP* nicotine vaping product, *SADs* smoking-attributable deaths, *SVADs* smoking and vaping attributable deaths, *LYLs* life-years lost, *FS-NVP* former smokers using NVPs

^a^Cumulative is the sum of SADs/SVADs or LYLs over the years 2013–2060

^b^No-NVP Scenario refers to the values of smoking prevalence (%), SADs, and LYLs in the absence of NVP use

^c^NVP Scenario refers to values of smoking prevalence (%), exclusive NVP prevalence (%), former smokers using NVP prevalence (%), SVADs, and LYLs with NVP use

In the No-NVP Scenario, smoking prevalence for ages 18 and above is 21.4% in 2013 declining to 17.4% in 2023, and 12.2% in 2060 for males, 15.9% in 2013 declining to 12.7% in 2023, and 8.7% in 2060 for females. Cumulative (2013–2060) SADs are 12.6 million for males and 5.0 million for females, while cumulative LYLs are 141.5 million for males and 54.1 million for females.

In the NVP Scenario, male (female) smoking prevalence declines to 12.9% (10.1%) in 2023 and then declines to 4.5% (4.1%) in 2060. Exclusive NVP users include those who initiated NVP use from never users (de novo) at all ages and those who switched to NVP use from smoking before age 35. Male (female) exclusive NVP use increases from 0% (0%) in 2013 to 3.8% (2.2%) in 2023, and to 8.8% (5.5%) in 2060. The prevalence of former smokers using NVPs (those switching from smoking to vaping after age 35) increases from 0% (0%) in 2013 to 1.5% (1.0%) in 2023, then increases to 1.6% (1.0%) in 2060. Compared with females, greater reduction in smoking and increase in NVP use in males is due to higher levels of switching to NVPs at younger ages among males. SVADs and LYLs cumulate to 11.3 million and 112.8 million during 2013–2060 for males and to 4.5 million and 43.9 million for females.

Under the NVP Scenario, approximately 1.8 million (1.3 million males, 0.5 million females) SVADs are averted, a 10.4% relative reduction compared with the No-NVP Scenario, and 38.9 million (28.7 million males, 10.1 million females) LYLs are averted, a 19.9% relative reduction.

### Sensitivity to NVP transition parameters

Sensitivity analyses are shown in Table 5 for smoking-attributable and vaping-attributable deaths (SVADs) and in Table 6 for life-years lost (LYLs). While holding the NVP relative risk multiplier (Risk_NVP_) constant at 5% (columns 2 and 3 in both tables), we examine the individual impact of variations in transition rates on SVADs and LYLs averted relative to the initial levels in the NVP Scenario. Each of five transition multipliers was varied for (1) NVP switching, (2) smoking initiation, (3) NVP initiation, (4) smoking cessation, and (5) NVP cessation.

**Table 5:** Sensitivity analysis: smoking-attributable deaths and deaths averted in the No-NVP Scenario and NVP Scenario across parameter changes, both genders, ages 18–99, 2013–2060

Scenario	NVP relative risk (Risk_**NVP**_)^**a**^ = 5%		NVP relative risk (Risk_**NVP**_) = 40%		Relative change (5% vs 40%)^d^
No-NVP Scenario	Total SADs		Total SADs		
	17,579,392	–	17,579,392	–	
NVP Scenario with parameter changes from baseline	Averted SADs and SVADs^b^	Relative change (vs. baseline estimate)^c^	Averted SADs and SVADs^b^	Relative change (vs. baseline estimate)^c^	
Baseline estimate^e^	1,833,727	0.0%	1,061,490	0.0%	− 42.1%
50% of switch rate,^f^ no decay^g^	1,124,559	− 38.7%	636,758	− 40.0%	− 43.4%
200% of switch rate, no decay	2,756,913	50.3%	1,611,641	51.8%	− 41.5%
100% of switch rate, 10% annual decay	1,102,703	− 39.9%	619,565	− 41.6%	− 43.8%
100% of switch rate, annually increase of 5% in the first 5 years	2,036,207	11.0%	1,182,961	11.4%	− 41.9%
25% of smoking initiation	1,938,925	5.7%	1,212,317	14.2%	− 37.5%
125% of smoking initiation multiplier	1,737,970	− 5.2%	924,283	− 12.9%	− 46.8%
25% of NVP initiation^i^	1,836,013	0.1%	1,104,549	4.1%	− 39.8%
75% of NVP initiation	1,831,576	− 0.1%	1,020,458	− 3.9%	− 44.3%
50% of smoking cessation^j^	224	− 100.0%	− 934,792	− 188.1%	− 41800%
150% of smoking cessation	2,913,448	58.9%	2,254,369	112.4%	− 22.6%
50% of NVP cessation^k^	1,782,054	− 2.8%	686,449	− 35.3%	− 61.5%
150% of NVP cessation	1,864,824	1.7%	1,291,320	21.7%	− 30.8%

*NVP* nicotine vaping product, *LYLs* life-years lost

^a^The NVP relative risk multiplier is the mortality risk of NVPs as a percentage of the excess mortality risk of smoking

^b^The absolute reduction in life-years lost in the NVP Scenario compared with the No-NVP Scenario over 2013–2060

^c^The relative percent change in averted LYLs for each NVP Scenario is compared with the initial NVP Scenario (best estimate). A negative (positive) value implies that changing the parameter will decrease (increase) the averted LYLs in the specific scenario relative to averted LYLs in the initial NVP Scenario

^d^The relative percent change in averted LYLs between scenarios with NVP risk multipliers of 5% vs. 40% is calculated as (Averted LYLs with 40% NVP risk − Averted LYLs with 5% NVP risk)/Averted LYLs with 5% NVP risk

^e^The initial values for each input parameter in the NVP Scenario are as follows. NVP switching rate with no decay for males females): 4% (2.5%) for ages 24 and below, 2.5% (2.0%) for ages 25–34, 2.5% (1.6%) for age 35–44, 1.3% (1.4%) for ages 45–54, 1.2% (1.4%) for ages 55–64, and 0.6% (1.0%) for ages 65 and above; smoking initiation multiplier = 75%; NVP initiation multiplier = 50%; Smoking cessation multiplier = NVP cessation multiplier =100%

^f^NVP switching rate is the annual rate at which current smokers switch to NVPs

^g^Annual decay rate is the exponential rate of decline in switching rates over time

^h^Smoking initiation multiplier is relative to smoking initiation in the No-NVP Scenario

^i^NVP initiation multiplier is relative to smoking initiation rates in the No-NVP Scenario

^j^Smoking cessation multiplier is relative to smoking cessation in the No-NVP Scenario

^k^NVP cessation multiplier is relative to smoking cessation rates in the No-NVP Scenario

**Table 6:** Sensitivity analysis: life-years lost and averted life years lost in the No-NVP Scenario and NVP Scenario across parameter changes, both genders, ages 18–99, 2013–2060

Scenario	NVP relative risk (Risk_NVP_)^**a**^ = 5%		NVP relative risk (Risk_NVP_) = 40%		Relative change (5% vs 40%)^d^
No-NVP Scenario	Total LYLs		Total LYLs		
	195,592,836	–	195,592,836	–	
NVP Scenario with parameter changes from baseline	Averted LYLs^b^	Relative change (vs. baseline estimate)^c^	Averted LYLs^b^	Relative change (vs. baseline estimate)^c^	
Baseline estimate^e^	38,866,015	0.0%	22,647,153	0.0%	− 41.7%
50% of switch rate,^f^ no decay^g^	24,568,915	− 36.8%	13,852,732	− 38.8%	− 43.6%
200% of switch rate, no decay	56,957,653	46.5%	33,756,316	49.1%	− 40.7%
100% of switch rate, 10% annual decay	23,976,479	− 38.3%	13,438,461	− 40.7%	− 44.0%
100% of switch rate, annually increase of 5% in the first five years	42,877,168	10.3%	25,119,390	10.9%	− 41.4%
25% of smoking initiation^h^	42,143,294	8.4%	27,380,768	20.9%	− 35.0%
125% of smoking initiation	35,915,686	− 7.6%	18,383,498	− 18.8%	− 48.8%
25% of NVP initiation^i^	38,925,863	0.2%	23,991,685	5.9%	− 38.4%
75% of NVP initiation	38,810,575	− 0.1%	21,372,002	− 5.6%	− 44.9%
50% of smoking cessation^j^	13,772,377	− 64.6%	− 5,035,938	− 122.2%	− 136.6%
150% of smoking cessation	55,337,637	42.4%	41,046,115	81.2%	− 25.8%
50% of NVP cessation^k^	38,048,498	− 2.1%	16,364,318	− 27.7%	− 57.0%
150% of NVP cessation	39,411,329	1.4%	26,857,771	18.6%	− 31.9%

*NVP* nicotine vaping product, *LYLs* life-years lost

^a^The NVP relative risk multiplier is the mortality risk of NVPs as a percentage of the excess mortality risk of smoking

^b^The absolute reduction in life-years lost in the NVP Scenario compared with the No-NVP Scenario over 2013–2060

^c^The relative percent change in averted LYLs for each NVP Scenario is compared with the initial NVP Scenario (best estimate). A negative (positive) value implies that changing the parameter will decrease (increase) the averted LYLs in the specific scenario relative to averted LYLs in the initial NVP Scenario

^d^The relative percent change in averted LYLs between scenarios with NVP risk multipliers of 5% vs. 40% is calculated as (Averted LYLs with 40% NVP risk − Averted LYLs with 5% NVP risk)/Averted LYLs with 5% NVP risk

^e^The initial values for each input parameter in the NVP Scenario are as follows. NVP switching rate with no decay for males females): 4% (2.5%) for ages 24 and below, 2.5% (2.0%) for ages 25–34, 2.5% (1.6%) for age 35–44, 1.3% (1.4%) for ages 45–54, 1.2% (1.4%) for ages 55–64, and 0.6% (1.0%) for ages 65 and above; smoking initiation multiplier = 75%; NVP initiation multiplier = 50%; Smoking cessation multiplier = NVP cessation multiplier = 100%

^f^NVP switching rate is the annual rate at which current smokers switch to NVPs

^g^Annual decay rate is the exponential rate of decline in switching rates over time

^h^Smoking initiation multiplier is relative to smoking initiation in the No-NVP Scenario

^i^NVP initiation multiplier is relative to smoking initiation rates in the No-NVP Scenario

^j^Smoking cessation multiplier is relative to smoking cessation in the No-NVP Scenario.

^k^NVP cessation multiplier is relative to smoking cessation rates in the No-NVP Scenario

If the baseline NVP switching rate parameter is reduced by 50% with 0% annual decay, NVP replacement of smoking is less in the NVP Scenario. Compared with the baseline NVP Scenario, averted SVADs decline in relative terms by 39% from 1.8 million to 1.1 million; averted LYLs decline by 37% from 38.9 million to 24.6 million. A doubling of the NVP switching rates from their initial levels yields a 50% relative increase in averted SVADs to 2.8 million and a 47% relative increase in averted LYLs to 57 million. Using the initial NVP switching rates but with a 10% decay rate reduces averted SVADs by 40% to 1.1 million and reduces averted LYLs by 38% to 24.0 million. Using the initial NVP switching rates but with a 5% annual increase rate in the first five years (2018–2022) increases averted SVADs by 11.0% to 2.0 million and increases averted LYLs by 10% to 42.9 million.

Increasing the smoking initiation multiplier from its baseline level primarily increases youth and young adult use at first, leading to more SVADs and LYLs later in life. An increase from 75 to 125% reduces averted SVADs by 5% and averted LYLs by 7.6%, and a reduction from 75 to 25%, increases averted SVADs by 6% and averted LYLs by 8%. Increasing or decreasing the NVP initiation multiplier yields a smaller relative change in averted SVADs and LYLs. Increasing the NVP initiation multiplier from 50% to 75% yields a relative reduction of 0.1% in averted SVADs and of 0.1% in averted LYLs. A reduction in the NVP initiation multiplier to 25% increases averted SVADs and averted LYLs by 0.2% or less.

Compared with changes in the initiation parameters, changes in cessation multipliers lead to a more immediate impact on SVADs, since cessation generally occurs later in life when mortality risk is highest. Increasing the smoking cessation multiplier from 100% to 150% of cessation in the No-NVP Scenario yields a 59% relative increase to 2.9 million averted SVADs and a 42% relative increase to 55.3 million averted LYLs. Reducing the smoking cessation multiplier from 100 to 50% yields a 100% relative reduction to 224 averted SVADs and a 64.6% relative reduction to 13.8 million averted LYLs. Comparable change in the NVP cessation multipliers yields less change. Increasing the NVP cessation multiplier from 100 to 150%, averted SVADs increased by 1.7% and LYLs increased by 1.4%. Reducing the NVP cessation multiplier to 50%, averted SVADs and LYLs decline by 2.8% and 2.1%, respectively.

Since the NVP risk is substantially lower than the smoking risk in the above analyses, the relative changes in averted SVADs and LYLs by varying the NVP initiation or cessation multipliers are less than the relative changes by varying the smoking initiation or cessation multipliers within comparable ranges.

### Sensitivity to NVP relative risks

Tables 5 and 6 also provide estimates of averted SVADs and LYLs with an NVP relative risk multiplier (Risk_NVP_) of 40% (columns 4 and 5) under the different NVP transition parameter ranges. With all other parameters at their baseline levels in the NVP Scenario, averted SVADs decrease in relative terms by 42% from 1.8 million with 5% NVP risks to 1.1 million with 40% NVP risks, and averted LYLs decrease by 42% from 38.9 million to 22.6 million.

By setting Risk_NVP_ at 40%, reducing the NVP switching rates by 50% yields a 39% relative reduction in averted LYLs, while increasing the NVP switching rates by 100% (from 100 to 200%) yields a 49% relative increase. Annually reducing the switching rates by 10% yields a 41% relative reduction in averted LYLs, while annually increasing the switching rates by 5% only in the first 5 years (2018–2022) yields an 11% relative increase in averted LYLs. Reducing the smoking initiation multiplier from 75 to 25% yields a 21% relative increase in LYLs, while an increase to 125% yields a 19% relative reduction compared with baseline. Reducing the NVP initiation multiplier from 50% to 25% yields 6% additional averted LYLs, while an increase to 75% yields 6% fewer averted LYLs. Reducing the smoking cessation multiplier from 100 to 50% yields 122% fewer averted LYLs, while an increase to 150% yields 81% additional averted LYLs. Reducing the NVP cessation rate multiplier from 100 to 50% yields 28% fewer averted LYLs, while increasing to 150% yields 19% additional averted LYLs.

Tables 5 and 6 (last row) also provide the change in SVADs and LYL with Risk_NVP_ at 40% compared with Risk_NVP_ = 5%. In all cases, the public health benefits in terms of averted SVADs and averted LYLs are reduced, with relative reductions of at least 23% associated with the NVP initiation and cessation rates as well as the smoking initiation and cessation rates. Thus, increasing NVP risks leads to greater sensitivity of LYLs to changes in switching, NVP initiation, and NVP cessation parameters.

## Discussion

The SAVM is the first simulation model incorporating vaping that was subjected to validation. SAVM projections of US smoking prevalence were generally close to estimates from 2013–2018 NHIS, except at younger ages where the relative reductions in smoking prevalence from surveys were greater than those predicted by the model. NVP use projections from SAVM were generally consistent with those of the 2018 NHIS.

While not discussed above, we also conducted validations of smoking prevalence against two other major national surveys: the Tobacco Use Supplement to the Current Population Survey (TUS-CPS) and the National Survey on Drug Use and Health (NSDUH) (See Supplement 3). The relative reductions in SAVM estimates were generally closer to 18–24-year-old estimates from these two surveys than to NHIS estimates, but they performed slightly less well overall. However, the prevalence estimates from TUS-CPS and NSDUH differ substantially from those of the NHIS both in terms of levels and trends over 2013–2018, which is consistent with previous studies [58, 81]. Thus, validation results may depend on the survey used to validate the model.

Using our baseline estimates of NVP relative mortality risks, switching rates, smoking and NVP initiation rates, and smoking and NVP cessation rates, the SAVM projects that NVP use in the population will translate to 1.8 million premature smoking-attributable and vaping-attributable deaths avoided and 38.9 million life-years gained from 2013–2060. However, these estimates are based on a specific set of parameters and should be carefully considered in the context of sensitivity analyses which show the influence of each parameter on public health outcomes. While the validation results increase confidence in the model, predictions for the future depend on the stability of its underlying transition behaviors and are subject to much greater uncertainty when considering longer time horizons.

The NVP relative risk relative to smoking was initially set to 5% [64, 75], but when increased to 40%, the associated public health gains were substantially reduced. This finding is particularly important in light of the uncertainty and controversy surrounding the relative risks of NVP use compared with smoking [25]. In addition, with the NVP relative risk at 5%, public health gains were more sensitive to the level of switching and smoking cessation rates and to a lesser extent smoking or NVP initiation rates. However, with NVP relative risks raised to 40%, public health impacts became much more sensitive to all parameters except for the NVP initiation rate.

Our analysis is subject to limitations. While our No-NVP Scenario is based on trends informed by data through 2013, future trends are subject to uncertainty and depend on tobacco control policies and other environmental changes that would have occurred if NVPs had not come onto the US market. The NVP relative risk, switching, initiation, and cessation parameters applied in the NVP Scenario are also subject to considerable uncertainty. Compared with smoking, NVPs are new products with less empirical evidence on their long-term health effects and usage/transition patterns [27, 37]. In addition, patterns of future NVP use are difficult to predict. NVP use is still relatively new and use patterns can be expected to change as a result of the “disruptive” nature of the product [1, 56]. Future NVP use will depend on product innovations and industry structure [56, 59, 60] and regulations surrounding NVP content, marketing, and use [51, 52]. In particular, NVP risks may be reduced with prudent FDA regulations that ban or limit the presence of known toxicants in e-liquids. In addition, public health benefits can be amplified with stronger policies directed at reducing smoking initiation and increasing smoking cessation.

Limitations to the model were identified in the 2013–2018 validation. In particular, the model underestimates the reduction in smoking among 18–24-year-olds observed in the NHIS, thereby overestimating future smoking prevalence. Because the No-NVP Scenario is based on age, period, and cohort effects through 2013, before NVPs became more widespread, some divergence is expected: more young adult smokers may be switching to NVP use or not initiating smoking than our model captures. Our sensitivity analyses indicated that public health impacts were particularly sensitive to the rate of switching from smoking to NVPs by 18–24-year-olds and smoking initiation in the NVP Scenario. In particular, downward trends in youth and young adult smoking have increased since 2017, when vaping devices such as JUUL became more widely used [15, 46, 63, 96]. The 2019 Monitoring the Future survey [66] indicates past 30-day smoking rates as low as 5.8% and daily smoking rates as low as 2%. Recent studies [2, 19, 58, 71, 97] also indicate that NVP use among US young adult smokers may be higher than indicated by our NVP initiation rates.

The validation of NVP use is sensitive to how regular NVP use is defined. Unlike cigarette smoking where regular use is generally defined in terms of having smoked at least 100 cigarettes during one’s lifetime, there is no accepted measure of regular NVP use. While we defined regular use by 10+ days in the last month, we also considered 1+ day and 20+ days measures based on PATH in 2013–2017. The 20+ days measure yielded levels similar to our 10+ days measure of NVP use, while the 1+ day measure yielded much higher estimates. Still, there is currently considerable uncertainty about NVP initiation, especially regarding initiation into more regular use [40, 67–69]. Although youth NVP rates increased substantially in 2019 [31, 100], they fell in 2020 [31], highlighting the challenges inherent in predicting future NVP use, even in the short-term. Nevertheless, like earlier simulation results [14, 73, 103], public health impacts were found to be relatively insensitive to the NVP initiation parameter.

Our NVP cessation multiplier baseline value of 100% implies that cessation from both smoking and NVPs is at the identical rate that of smoking cessation in the No-NVP Scenario. This transition implicitly includes cessation from both smoking and vaping. While this complete cessation from smoking may increase with the availability of NVPs, some smokers may instead switch to NVPs. In our model, the public health impact regarding smoking depends on the number who quit smoking through both complete cessation and switching in the NVP Scenario compares to the number of smokers who would otherwise quit in the absence of NVPs (the No-NVP Scenario). In addition, some recent evidence indicates that NVPs may be less addictive than smoking [7, 61, 62, 72, 87], implying a value greater than 100% and that those smokers switching to vaping may be more likely to quit vaping in future years than smokers in the No-NVP Scenario.

The NVP Scenario in SAVM applies simplifying assumptions so that the model is user-friendly. A never smoker who experiments with NVPs and does not transition to smoking is considered a non-user*.* Because transitions to and from dual use are often particularly unstable [4, 8, 18, 78], we have chosen not to distinguish dual from exclusive cigarette use in the NVP Scenario. In addition, consistent with previous CISNET models [43–45], SAVM does not consider relapse, with transitions to former smokers or former NVP users treated as permanent. Except for the switching parameter, we have assumed that the NVP relative risk and other transition parameters are age and time invariant to simplify the analysis. However, other key parameters, such as the smoking cessation and initiation parameters could shift over time. Intertemporal and age patterns will depend on technological advances in NVPs and regulatory policies. It will be important to update key parameters to reflect new evidence as it becomes available.

Finally, although other categories of nicotine delivery products are available on the market, including cigars, smokeless tobacco, and heated tobacco products [21], we only consider two categories of products, cigarettes, and NVPs, to keep the model tractable. As a substitute for NVPs, heated tobacco products in particular may affect NVP as well as cigarette use.

While subject to limitations, the SAVM model can be easily modified. The model is developed in Excel both for transparency and to ensure its accessibility to non-technical audiences. More experienced users can modify the model’s underlying assumptions and structure as described in the SAVM User Guide, thereby providing the user flexibility. The model and an accompanying user guide are available at https://tcors.umich.edu/Resources_Download.php?FileType=SAV_Model.

The SAVM can also be easily applied to other contexts and policy scenarios. The requisite data on smoking prevalence and population by age and gender are generally available by state and for most countries. Further, the model can be used to consider the impact of different policies by comparing the NVP Scenario under current conditions to a scenario with user-specified policy parameters. However, the results will depend on the user’s knowledge of the impact of the policy both on cigarette and NVP use and any age- or cohort-related variations in these parameters, so that sensitivity analysis is strongly encouraged.

In conclusion, the SAVM was developed as a user-friendly tool to examine the potential public health impact of NVP use on smoking behaviors and to show how public health outcomes vary across different assumptions related to NVP health risks and the initiation and cessation of NVP use and smoking. Using readily available data, SAVM can be applied by policymakers, researchers, and other stakeholders in other countries to help understand the role of NVPs vis-à-vis smoking and the impact that those products have on public health.

## Supplementary Information

**Additional file 1: Supplement 1.** Mathematical formulation of the SAVM model. **Supplement 2.** Estimation of the Switch Rate from Cigarette to NVP Use. **Supplement 3.** Further validation of the model.

## Data Availability

The model, data used therein, and a 100-page user manual will be made available to all requestors, and we have made the model available on our (University of Michigan) FDA Tobacco Center of Regulatory Science (TCORS) website at https://tcors.umich.edu/Resources_Download.php?FileType=SAV_Model.

## Abbreviations

NVP: Nicotine vaping product
SAVM: Smoking and Vaping Model
SADs: Smoking-attributable deaths
LYLs: Life years lost
