# Supplementary material for: Public health implications of vaping in the USA: the smoking and vaping simulation model
Source: Popul Health Metr. 2021 Apr 17;19:19. doi: 10.1186/s12963-021-00250-7 (PMC8052705; doi:10.1186/s12963-021-00250-7)
Supplement: Supplementary file 1 — Additional file 1: Supplement 1. Mathematical formulation of the SAVM model. Supplement 2. Estimation of the Switch Rate from Cigarette to NVP Use. Supplement 3. Further validation of the model. [file 12963_2021_250_MOESM1_ESM.docx]

**Supplement 1. Mathematical formulation of the SAVM model**

**The No-NVP Scenario**

The No-NVP (nicotine vaping product, aka e-cigarettes) Scenario projects the prevalence of current, and former smokers over time using age- and gender-specific initiation and cessation rates and age-, gender-, and smoking status-specific mortality rates for each cohort. Never smokers are denoted as never users in the No-NVP Scenario.

For each cohort, the projected prevalence of never users at age a and year t is composed of the surviving never users in the previous year (age a-1, year t-1) who did not initiate smoking. Denoting the initiation rate as *Init_a-1,t-1_* and the death rate of never users in the previous year as *DR_never_a-1,t-1_*, never user prevalence at age a and year t is calculated as:

Prev_never_a,t_= Prev_never_a-1,t-1_ * (1 - DR_never_a-1,t-1_) * (1 – Init_a-1,t-1_). (1)

The prevalence of current smokers includes surviving never users who initiate smoking and surviving current smokers who do not quit. Denoting the permanent cessation rate from smokers at a-1 and t-1 to former smokers at a and t as *Cess_a-1,t-1_* and the smoker death rate as *DR_smoker_a-1,t-1_*, current smoking prevalence evolves as:

Prev_smoker_a,t_ = Prev_smoker_a-1,t-1_ * (1- DR_smoker_a-1,t-1_) * (1- Cess_a-1,t-1_)

+ Prev_never_a-1,t-1_ * (1 - DR_never_a-1,t-1_) * Init_a-1,t-1_. (2)

Denoting *DR_former_a,t_* as the former smoker death rate at a and year t, former smokers include surviving former smokers and surviving smokers who permanently quit smoking. Former smoking prevalence is:

Prev_former_a,t_ = Prev_former_a-1,t-1_*(1–DR_former_a-1,t-1_)+Prev_smoker_a-1,t-1_

*(1- DR_smoker_a-1,t-1_) * Cess_a-1,t-1_. (3)

Since prevalence estimates decline each year due to deaths, each of the prevalence rates is multiplied by the following correction factor to assure that they sum to 100%:

Correction factor_a,t_ = 1/[Prev_never_a-1,t-1_*(1-DR_never_a-1,t-1_) + Prev_smoker_a-1,t-1_*

(1-DR_smoker_a-1,t-1_) + Prev_former_a-1,t-1_*(1-DR_former_a-1,t-1_)]. (4)

**The NVP Scenario**

Starting from the same initial current, former and never smoking prevalence as in the No-NVP Scenario, the NVP Scenario is expanded to include current and former NVP use.

The model directly relates the NVP Scenario to the No-NVP Scenario by defining the NVP and smoking initiation rates in the NVP Scenario through separate linear multipliers applied to the smoking initiation rate (*Init_a.t_*) in the No-NVP Scenario. Similarly, NVP and smoking cessation are modelled as separate linear multipliers of smoking cessation (*Cess_a,t_*) in the No-NVP Scenario, so that cessation follows the same age pattern in the No-NVP Scenario and tends to increase with age. A switching parameter (σ_a_) with an annual decay rate (δ) is defined that allows direct transitions from smoking to vaping.

Never users include surviving never users who do not initiate into smoking or NVP use, calculated as:

Prev_never_a,t_ = Prev_never_a-1,t-1_ *(1 – DR_never_a-1,t-1_)* (1 – α_S_* Init_a-1,t-1_– α_V_ * Init_a-1,t-1_), (5)

where *α_S_* and *α_V_* are the smoking (S) and vaping (V) initiation multipliers.

Smokers include surviving smokers who do not switch to vaping and who do not quit smoking and surviving never users who initiate smoking:

Prev_smoker_a,t_ = Prev_smoker_a-1,t-1_*(1-DR_smoker_a-1,t-1_)*(1 – σ_a-1_*(1-δ)^t-t0-1^ – χ_S_*

Cess_a-1,t-1_) + Prev_never_a-1,t-1_ * (1–DR_never_a-1,t-1_) *(α_S_*Init_a-1,t-1_), (6)

where *σ_a-1_* is the switch rate from smoking to NVP use in the initial year t_0_ with decay rate *δ* for each cohort and χ*_S_* is the smoking cessation multiplier. We note that smokers include dual users of cigarettes and NVPs.

Exclusive NVP users include surviving never users who initiate vaping, surviving vapers who do not quit, and those switching to vaping from surviving current smokers before age 35:

Prev_NVP_a,t_ = Prev_never_a-1,t-1_*(1-DR_never_a-1,t-1_)*α_V_*Init_a-1,t-1_ + Prev_NVP_a-1,t-1_*

(1-DR_NVP_a-1,t-1_)*(1– χ_V_ * Cess _a-1,t-1_) + Prev_smoker_a-1,t-1_* I(a-1<35)*

(1–DR_smoker_a-1,t-1_)* σ_a-1_*(1-δ)^t-t0-1^_,_ where I(a-1<35)=1 if a<36, 0 otherwise. (7)

*DR_NVP* is the death rate of exclusive NVP users and χ*_V_* is the NVP cessation multiplier. In this formulation, those who quit smoking before age 35 are treated the same as never smokers who vape rather than former smokers, because mortality risks of smoking are minimal when quitting before age 35.

Former smokers include surviving former smokers, surviving smokers who quit smoking and do not vape, and surviving former smokers who regularly vaped but quit vaping:

Prev_former_a,t_= Prev_former_a-1,t-1_*(1–DR_former_a-1,t-1_) +Prev_smoker_a-1,t-1_*(1–DR_smoker_a-1,t-1_)*

(χ_S_* Cess _a-1,t-1_) + Prev_FS-NVP_a-1,t-1_*(1 – DR_FS-NVP_a-1,t-1_) * (χ_V_* Cess _a-1,t-1_), (8)

where Prev_FS-NVP_a-1,t-1_ is the prevalence of former smokers who currently use NVPs and DR_FS-NVP_a-1,t-1_ is the corresponding death rate.

Former NVP users include surviving former NVP users and surviving vapers who quit vaping:

Prev_FNVP_a,t_ = Prev_FNVP_a-1,t-1_ * (1- DR_FNVP_a-1,t-1_) +Prev_NVP_a-1,t-1_ * (1 – DR_NVP_a-1,t-1_) * (χ_V_ * Cess_a-1,t-1_)_,_  (9)

where *DR_FNVP_a-1,t-1_* denotes the former NVP user death rate.

Since former smokers using NVPs may quit vaping, former smokers using NVPs includes surviving former smokers using NVPs who do not quit vaping and smokers who switch to vaping (after age 35):

Prev_FS-NVP_a,t_ = Prev_FS-NVP_a-1,t-1_*(1- DR_FS-NVP_a-1,t-1_)*(1 – χ_V_ * Cess _a-1,t-1_)

+ Prev_smoker_a-1,t-1_* I(a-1$\geq$35) * (1- DR_smoker_a-1,t-1_)*(σ_a-1_*(1-δ)^t-t0-1^),

where I(a-1$\geq35$) =1 if a>36, 0 otherwise. (10)

As in the No-NVP Scenario, a correction factor is applied in the NVP Scenario, which equals to the reciprocal of the sum of prevalence rates for each smoking and vaping category i to ensure that the prevalence rates each period sum to 100%:

Correction factor_a,t_ = 1 / ∑_i_ [Prev_i_a-1,t-1_ * (1 – DR_i_a-1,t-1_)]. (11)

**The NVP Scenario**

Starting from the same initial current, former and never smoking prevalence as in the No-NVP Scenario, the NVP Scenario is expanded to include current and former NVP use.

A switching parameter allows direct transitions from smoking to vaping. The model directly relates the NVP Scenario to the No-NVP Scenario by defining the NVP and smoking initiation rates in the NVP Scenario through separate linear multipliers applied to the smoking initiation rate (*Init_a.t_*) in the No-NVP Scenario. Similarly, NVP and smoking cessation are modelled as separate linear multipliers of smoking cessation (*Cess_a,t_*) in the No-NVP Scenario, so that cessation follows the same age pattern in the No-NVP Scenario and tends to increase with age.

Never users include surviving never users who do not initiate into smoking or NVP use, calculated as:

Prev_never_a,t_ = Prev_never_a-1,t-1_ *(1 – DR_never_a-1,t-1_)* (1 – α_S_* Init_a-1,t-1_– α_V_ * Init_a-1,t-1_), (5)

where *α_S_* and *α_V_* are the smoking (S) and vaping (V) initiation multipliers.

Smokers include surviving smokers who do not switch to vaping and who do not quit smoking and surviving never users who initiate smoking:

Prev_smoker_a,t_ = Prev_smoker_a-1,t-1_*(1-DR_smoker_a-1,t-1_)*(1 – σ_a-1_*(1-δ)^t-t0-1^ – χ_S_* Cess_a-1,t-1_)

+ Prev_never_a-1,t-1_ * (1–DR_never_a-1,t-1_) *(α_S_*Init_a-1,t-1_), (6)

where *σ_a-1_* is the switching rate from smoking to NVP use in the initial year t_0_ with decay rate *δ* for each cohort and χ*_S_* is the smoking cessation multiplier. We note that smokers include dual users of cigarettes and NVPs.

Exclusive NVP users include surviving never users who initiate vaping, surviving vapers who do not quit, and those switching to vaping from surviving current smokers before age 35:

Prev_NVP_a,t_ = Prev_never_a-1,t-1_*(1-DR_never_a-1,t-1_)*α_V_*Init_a-1,t-1_ + Prev_NVP_a-1,t-1_*

(1-DR_NVP_a-1,t-1_)*(1– χ_V_ * Cess _a-1,t-1_) + Prev_smoker_a-1,t-1_* I(a-1<35)*

(1–DR_smoker_a-1,t-1_)* σ_a-1_*(1-δ)^t-t0-1^_,_ where I(a-1)=1 if a<36, otherwise = 0. (7)

*DR_NVP* is the death rate of exclusive NVP users and χ*_V_* is the NVP cessation multiplier. In this formulation, those who quit smoking before are treated the same as never smokers who vape rather than former smokers, because mortality risks of smoking are minimal when quitting before age 35.

Former smokers include surviving former smokers, surviving smokers who quit smoking and do not vape, and surviving former smokers who regularly vaped but quit vaping:

Prev_former_a,t_= Prev_formers_a-1,t-1_*(1–DR_former_a-1,t-1_) +Prev_smoker_a-1,t-1_*(1–DR_smoker_a-1,t-1_)*

(χ_S_* Cess _a-1,t-1_) + Prev_FS-NVP_a-1,t-1_*(1 – DR_FS-NVP_a-1,t-1_) * (χ_V_* Cess _a-1,t-1_), (8)

where Prev_FS-NVP_a-1,t-1_ is the prevalence of former smokers who currently use NVPs and DR_FS-NVP_a-1,t-1_ is the corresponding death rate.

Former NVP users include surviving former NVP users and surviving vapers who quit:

Prev_FNVP_a,t_ = Prev_FNVP_a-1,t-1_ * (1- DR_FNVP_a-1,t-1_) +

Prev_NVP_a-1,t-1_ * (1 – DR_NVP_a-1,t-1_) * χ_V_ * Cess_a-1,t-1,_  (9)

where *DR_FNVP_a-1,t-1_* denotes the former NVP user death rate.

Since former smokers using NVPs may quit vaping, former smokers using NVPs (after age 35) includes surviving former smokers using NVPs who do not quit vaping and smokers who switch to vaping:

Prev_FS-NVP_a,t_ = Prev_FS-NVP_a-1,t-1_*(1- DR_FS-NVP_a-1,t-1_)*(1 – χ_V_ * Cess _a-1,t-1_)

+ Prev_smoker_a-1,t-1_* I(a-1>35) * (1- DR_smoker_a-1,t-1_)*(σ_a-1_*(1+δ)^t-t0-1^),

where I(a-1) =1 if a>36, otherwise = 0 (10)

As in the No-NVP Scenario, a correction factor is applied as the reciprocal of the sum of prevalence rates for each smoking and vaping category i to ensure that the prevalence rates each period sum to 100%:

Correction factor_a,t_ = 1 / ∑_i_ [Prev_i_a-1,t-1_ * (1 – DR_i_a-1,t-1_)]. (11)

**Public Health Outcomes**

The SAVM considers two public health outcomes considered: 1) Smoking- and vaping-attributable deaths, and 2) smoking- and vaping-attributable life-years lost (LYLs).. Both are based on the excess risks of smoking or vaping and the number of current and former smokers and vapers.

In the No-NVP Scenario, smoking-attributable deaths (SADs) for current smokers is calculated by applying the excess risks of smokers relative to never smokers (DR_smoker_a,t_ – DR_never_a,t_) to the smoking population:

SAD_smoker_a,t_ = (DR_smoker_a,t_ – DR_never_a,t_) * Prev_smoker_a,t_ * Population_a,t_, (12)

and attributable deaths for former smokers is calculated as:

SAD_former_a,t_ = (DR_former_a,t_ – DR_never_a,t_) * Prev_former_a,t_ * Population_a,t_ (13)

SADs for current and former smokers are summed over all ages in a particular year to obtain total SADs in that year. LYLs are estimated as the number of premature deaths multiplied by the remaining life expectancy (LE) of a never smoker at each age in year t denoted by LE_Never_a,t_:

LYL_a,t_ = (SAD_smoker_a,t_ + SAD_former_a,t_ ) * LE_Never_a,t._ (14)

Summing over ages in a particular year obtains the LYLs in that year.

In the NVP Scenario, SAD_smoker_a,t_ and SAD_former_a,t_ are calculated in the same manner as above except using their respective prevalence rates in the NVP scenario. Vaping-attributable deaths for current exclusive NVP users (VADs) and for former NVP users (FNVP) are measured by two similar equations (15) and (16). For current exclusive NVP users, attributable deaths (VAD_NVP_a,t_) are a product of the number of NVP users and the excess risks of smoking adjusted by NVP relative risk, denoted by Risk_NVP_, and similarly for former NVP use attributable deaths (VAD_FNVP_a,t_):

VAD_NVP_a,t_ = Risk_NVP_*(DR_smoker_a,t_ - DR_never_a,t_)*Prev_NVP_a,t_*Population_a,t._ (15)

VAD_FNVP_a,t_ = Risk_NVP_*(DR_former_a,t_ -DR_never_a,t_)*Prev_FNVP_a,t_*Population_a,t. (_16)

As a special case in the NVP Scenario, the death rate of NVP users who previously smoked is determined by the portion of excess risk of current relative to former smokers plus the death rate of former smokers:

DR_FS-NVP_a,t_ *=* Risk_NVP_ ** (*DR_smoker_a,t_ - DR_former_a,t_*) +* DR_former_a,t_ (17)

Smoking and vaping-attributable deaths (SVADs) for former smokers who currently use NVPs (FS-NVPs) in the NVP Scenario is measured as a product of the number of FS-NVPs and the excess mortality risk of FS-NVP to never smokers:

SVAD_FS-NVP_a,t_ = [*DR_FS-NVP_a,t_* -DR_never_a,t_)]*Prev_FS-NVP_a,t_* Population_a,t_ (18)

Attributable deaths are calculated by summing SADs, VADs and SVADs over ages each year for a given cohort. LYLs in the NVP Scenario at each age are calculated as:

LYL_a,t_ = (SAD_smoker_a,t_ + SAD_former_a,t_ + VAD_NVP_a,t_ + VAD_FNVP_a,t_ +

SVAD_FS-NVP_a,t_)*LE_Never_a,t_ (19)

Summing over ages in a particular year obtains the LYLs in that year.

The public health impact of NVP use each year is evaluated as the difference in attributable deaths between the No-NVP and NVP Scenarios, and similarly for LYLs.

**Supplement 2.** **Estimation of the Switch Rate from Cigarette to NVP Use**

In this supplement, we discuss the methodologies used to estimate the one-year switch rate from dual or exclusive smoking to regular NVP (nicotine vaping product, aka e-cigarettes) use. Switch rates are measured in terms of those who quit smoking in the last year and who exclusively use NVPs. This measure does not include those that may have used NVPs to quit both smoking and NVP use in the last year.

We used data from the Population Assessment of Tobacco and Health (PATH) Study to calculate the annual switch rates from smokers to exclusive NVP users. We use the four waves (wave 1 in 2013/2014 to wave 4 in 2016/2017), prior to the growth in Juul use. Current smokers are defined as those who have smoked 100 cigarettes lifetime and currently smoke every day or some days. We do not distinguish dual users of e-cigarettes and cigarettes from exclusive cigarette smokers. Former smokers include those who quit less than 1 year. To measure NVP use, we use current e-cigarette use in PATH waves 1 and 2, since questions are in terms of “e-cigarettes.” In waves 3 and 4, we use current information on all e-products, defined as e-cigarette, e-pipe, e-hookah, e-cigar, and other electronic vaping products.

Our goal is to distinguish transitions to “regular” exclusive NVP use, that are relatively stable over time. To help ensure regular use, we initially considered only those ever users that currently use NVPs “fairly regularly.” Because more frequent users are less likely to be experimental use and thus more likely to continue use, we consider four frequency of use measures, e.g., at least 1 day, at least 5 days, at least 10 days and at least 20 days in the past 30 days. We consider two different types of measures: 1) prospective using individual longitudinal transitions, and 2) retrospective using implied transitions based on changes in population use patterns over time.

**Method 1. Prospective Measure**

Our prospective measure considers longitudinal transitions of smokers wave t to exclusive NVP users at wave t+1. With four waves available, we estimated three switch rates: wave 1 to wave 2, wave 2 to wave 3, and wave 3 to wave 4. The age-specific switch rate is based on the age in the prior wave. The switch rate is calculated as a percentage of the former smokers using NVPs exclusively this year (t+1) who transitioned from being smokers last year (t) among those who were smokers last year (t), i.e.,

Switch_rate(t+1) = [Former smokers exclusively using NVPs at t+1 who were smokers at t] / [Former smokers at t+1 who were smokers at t + Current smokers at t+1 who were smokers at t].

In examining the transitions over time, three issues were considered:

1. Missing values, those observations with unknown smoking status at t may have information for smoking and NVP using status at t+1.

2. Lost data, those observations with known smoking status at t may have unknown smoking or NVP using status at t+1.

3. Inconsistent cases, those observations with known smoking status at t and known smoking and NVP using status at t+1, but the reported status is inconsistent between two time periods. E.g. smokers at t reported they had been former smokers for more than 2 years at t+1.

This method only considers individuals who were known smokers at t, so the first issue can be avoided. The observations with unknown NVP status at t+1 (issue 2) were omitted. Former smokers with either unknown or inconsistent length of quitting at t+1 were either included or excluded in the analyses, as described below.

***Definition 1: Former smokers based on smoking status one year ago***

In our first set of analyses, the former smokers were defined as former smokers at wave t+1 and current smokers at wave t. We did not consider information provided in the survey regarding when they quit. The estimates of switch rates and relative changes in those rates between waves are provided in Table S2.1 and Table S2.2, respectively.

Using regular NVP use defined as 1+ days use in the past-30 days, the unweighted switch rate for male (female) smokers is 2.4% (1.5%) from wave 1 to 2, increasing to 2.5% (1.5%) from wave 2 to 3 and declining to 1.9% (1.4%) from wave 3 to 4. The relative increase in switch rates in wave 2 vs wave 1 is 3.3% (0.4%) and the relative reduction in wave 3 vs wave 2 is 22.6% (6.8%).

Using regular NVP use defined as 10+ days use in the past-30 days, the unweighted switch rate is 2.2% (1.4%) from wave 1 to 2, 2.2% (1.2%) from wave 2 to 3, and 1.6% (1.3%) from wave 3 to 4. The relative increase in switch rates for males in wave 2 vs wave 1 is 1.0% and the relative reduction in wave 3 vs wave 2 is 27.5%. The switch rates for females decreased by 13.0% in wave 2 vs wave 1 but slightly increased by 6.0% in wave 3 vs wave 2.

Using regular NVP use defined as 20+ days use in the past-30 days, the unweighted switch rate is 2.1% (1.3%) from wave 1 to 2, 2.0% (1.1%) from wave 2 to 3, and 1.5% (1.2%) from wave 3 to 4. The relative reduction in switch rates for males in wave 2 vs wave 1 is 6.8% and 22.4% in wave 3 vs wave 2. The switch rates for females decreased by 10.8% in wave 2 vs wave 1 but increased by 8.2% in wave 3 vs wave 2.

There are 7 age groups in waves 1, 2, and 3, i.e. age 18-24, 25-34, 35-44, 45-54, 55-64, 65-74, and 75 and above. Due to small sample size of the age group 75 and above, we excluded this group in analyzing the range and pattern of transitions for both genders. The transitions in all waves mainly occurred before age 45.

As a gauge of the general levels, we average rates over the three sets of transitions in Supplement 2: Table7 with the label “Prospective Def 1”.

***Definition 2:* *A stricter definition for selected former smokers***

The analysis above did not specifically consider years since quit smoking in the definition of former smokers, and we considered a stricter definition for former smokers in the second analyses. As a stricter definition, former smokers were defined to meet the criterion that they were former smokers who quit less than two years at wave t+1 and were current smokers at wave t. This method eliminates observations with inconsistent answers. The estimates of switch rates and relative changes in those rates between waves are provided in Table S2.3 and Table S2.4, respectively.

Using regular NVP use defined as 1+ days use in the past-30 days, the unweighted switch rate is 2.4% (1.5%) for male (female) smokers from wave 1 to 2, decreasing to 2.3% (1.5%) from wave 2 to 3 and 1.7% (1.3%) from wave 3 to 4. The relative reduction in switch rates for males (females) in wave 2 vs wave 1 is 3.0% (4.6%) and in wave 3 vs wave 2 is 28.1% (9.0%) (Table S2.4).

Using regular NVP use defined as 10+ days use in the past-30 days, the unweighted switch rate is 2.2% (1.4%) for male (female) smokers from wave 1 to 2, decreasing to 2.1% (1.2%) from wave 2 to 3 and 1.4% (1.2%) from wave 3 to 4. The relative reduction in switch rates for males is 4.8% in wave 2 vs wave 1and 33.7% in wave 3 vs wave 2. The switch rates for females decreased by 18.3% in wave 2 vs wave 1 but slightly increased by 4.2% in wave 3 vs wave 2.

Using regular NVP use defined as 20+ days use in the past-30 days, the unweighted switch rate is 2.2% (1.3%) for male (female) smokers from wave 1 to 2, decreasing to 1.9% (1.1%) from wave 2 to 3 and 1.4% (1.1%) from wave 3 to 4. The relative reduction in switch rates for males is 10.5% in wave 2 vs wave 1 and 29.3% in wave 3 vs wave 2. The switch rates for females decreased by 16.7% in wave 2 vs wave 1 but slightly increased by 6.4% in wave 3 vs wave 2.

The transitions again mainly occurred before age 45. To gauge general levels, we average rates over the three sets of transitions. The results are shown in Supplement 2: Table7 with the label “Prospective Def 2”.

Switch rates are generally slightly higher using the less strict definition of former smokers, especially in the later waves. However, the results in both analyses using different definition of former smokers are quite similar.

**Method 2. Retrospective Measure**

When calculating the switch rates by the retrospective method, we use only cross-sectional data to consider those smokers one year ago. The current smokers and former smokers who have quit less than one year at wave t+1 are assumed to be current smokers at t. Given four waves in PATH, we estimated the switch rate for each wave. The switch rate is measured as a percent of those who are exclusive NVP users and have quit smoking less than one year at wave t among those who are smokers along with those who have quit less than one year at wave t. We note that this may create a bias, particularly at younger ages, because it includes those that initiated smoking in the last year, but were not smokers one year ago. When calculating the switch rates by the retrospective method, the following formula is applied:

Switch_rate (t) = [Former smokers who quit less than one year and currently use NVPs exclusively at t] / [Former smokers who quit less than one year at t + current smokers at t].

The estimates of switch rates and relative changes in those rates between waves are provided in Table S2.5 and Table S2.6, respectively.

Using regular NVP use defined as 1+ days use in the past-30 days, the unweighted switch rate is 1.3% (1.6%) for male (female) smokers in wave 1, increasing to 2.8% (1.8%) in wave 2, 3.1% (1.9%) in wave 3, and 3.3% (2.0%) in wave 4. The relative increase in switch rates is 107.2% (14.1%) from wave 1 to 2, 12.8% (4.9%) from wave 2 to 3, and 5.6% (1.3%) from wave 3 to 4 for males (females).

Using regular NVP use defined as 10+ days use in the past-30 days, the unweighted switch rate is 1.3% (1.6%) for male (female) smokers in wave 1, increasing to 2.4% (1.6%) in wave 2, 2.5% (1.4%) in wave 3, and 2.7% (1.6%) in wave 4. The relative change in switch rates is 89.9% (-0.1%) from wave 1 to 2, 5.4% (-9.5%) from wave 2 to 3, and 7.6% (11.8%) from wave 3 to 4 for males (females).

Using regular NVP use defined as 20+ days use in the past-30 days, the unweighted switch rate for male smokers is 1.2% in wave 1, increasing to 2.2%-2.5% for waves 2-4, and is 1.5% for female smokers in wave 1, decreasing to 1.4% and 1.2% in wave 2-3 but increasing to 1.5% in wave 4. The relative change in switch rates is 86.4% (-8.1%) from wave 1 to 2, 1.5% (-9.3%) from wave 2 to 3, and 12.1% (18.9%) from wave 3 to 4 for males (females).

There are 7 age groups in waves 1, 2, and 3, i.e. age 18-24, 25-34, 35-44, 45-54, 55-64, 65-74, and 75 and above. However, there are 6 age groups in wave 4, i.e. age 18-24, 25-34, 35-44, 45-54, 55-64, 65 and above. Due to small sample size of the age group 75 and above, we excluded this group in analyzing the range and pattern of switching. The last age group in wave 4 was compared with 65-74 age group from other waves. Because the rates generally increased substantially from wave 1 to 2, we average rates over the last 3 waves, and the results are shown as “Retrospective” in Table S2.7.

The retrospective measures generally yield higher estimated transitions in wave 2 than the prospective measure in going from wave 1 to 2, and, unlike the prospective measure show slightly increasing transitions in later waves. In general, it is difficult to conclude that there is the same pattern for switch rates over time by different measures.

**Sensitivity Analysis Regarding Weighted vs. Unweighted Measures**

Above we use unweighted measures to avoid any bias from applying weights of a selected sample. As a sensitivity analysis, we estimated switch rates incorporating weights. Compared with unweighted switch rates, most of the weighted rates were slightly lower for males and slightly higher for females, but in general the results are very similar between two rates. Using the prospective methods and retrospective method, the differences between the two kinds of rates for adult males and females are less than 20% in relative terms. We only show the weighted average switch rates over waves in Table S2.8, which can be compared to the unweighted results shown in Table S2.7.

We also considered estimates of NVP use based on the measure of “regular past-30-days use” and the measure of “regular every day and somedays use”. For the regular past-30-day use measure, the key questions asked for ever NVP users are “(1) In the past 30 days, have you used an electronic nicotine product, even one or two times”, “(2) When did you last use an electronic nicotine product”, “(3) About how long has it been since you last took a puff from an electronic nicotine product”, “(4) On how many of the past 30 days did you use an electronic nicotine product”, and “(5) Have you ever used electronic nicotine products fairly regularly?” The regular ever NVP users who used any NVP in the past 30 days are defined as past-30-day users, and differs from the definition based on the 1+ days of NVP use in the past-30 day above due to missing values (some past-30-day users may have not answered question (4)) and inconsistent answers (some past-30-day users may answer that they used NVPs 0 day in past 30 days). In PATH codebooks, the variables for past-30-day NVP users are provided for all waves. Therefore, we estimate the switch rates for past-30-day users of NVP. Compared with our estimates based on 1+ days of NVP use in the past-30 day in the previous sections, the measure for the past-30-day users provides more NVP users and higher switch rates. Using the past-30-day use definition for regular NVP use, the relative difference in the average unweighted switch rates between waves is within the range of 0% to 13% for males and -1% to 13% for females at all age groups.

The regular every day and someday use measure is based on the questions of ever NVP users: “(1) Do you now use electronic nicotine products every day or some days?” and “(2) Have you ever used electronic nicotine products fairly regularly?” The regular ever users who currently use NVPs every day or some days are defined as the established current users. In PATH codebooks, the variables for established current NVP users are provided for all waves, which were used to estimate switch rates. the variables for established current NVP users are provided for wave 1 and 2 and the variables for established current NVP users are provided for wave 2, 3 and 4. Therefore, we estimate the switch rates for NVP use in wave 1 and for NVPs in waves 2, 3, and 4. Compared with our estimates based on 1+ days of NVP use in the past-30 day in the previous section, the measure for “every day or some days” users provides similar NVP users and switch rates. Using the “every day or some days” definition for regular NVP use, the relative difference of the average unweighted switch rates between waves is within the range of -6% to 2% for males and -6% to 9% for females at all age groups. Further sensitivity analysis may consider:

1. Those NVP users who quit $\leq1$ year instead of just < 1 year to check of whether there is a sensitivity to those who have rounded to one year.
2. Non-regular use to consider those who do not consider themselves regular users, although using $\geq10$ days.
3. Only smokers that initiated NVPs at least one year ago.

**Final Estimates**

Based on the weighted and unweighted average transitions rates in Table S2.7 and 8, we provide our “best estimates” of the transitions from smoking to exclusive NVP use by age and gender. In developing the estimates, we focused most closely on the prospective unweighted estimates using the regular NVP use defined as 10+ days use in the past-30 day for each of the age ranges. However, we also considered neighboring age ranges, the range of rates over different NVP measures (at least 1, 5, 10, or 20 of the past 30 days) and three estimation methods (two prospective methods and the retrospective method). The Table S2.9 show the best estimates for switch rates by age group and gender, with ranges covered all the other estimates from the analyses by using various definition of regular NVP use and estimation methods. These best estimates for switch rates by age group and gender were applied in our SAVM model.

The switch rates generally decline with age for adult males and females. Before age 45, males have higher rates than females. For ages 45-54, the rates for males and females are close to each other. After age 54 (exclude ages 75+), females have slightly higher rates than males. For ages 18-24, our best estimates of switch rates are 4.0% with a range (3.4%-4.8%) for males and 2.5% with a range of (1.7%-3.1%) for females. For ages 25-34, the best estimates of switch rates are 2.5% with a range of (2.2%-3.1%) for males and 2.0% with a range of (1.6%-2.2%) for females. For ages 35-44, the best estimates are 2.5% with a range of (1.4%-2.5%) for males and 1.6% with a range of (1.0%-1.8%) for females. For ages 45-54, the best estimates of switch rates are 1.3% with a range of (0.9%-1.3%) for males and 1.4% with a range of (0.8%-1.4%) for females. For ages 55-64, the best estimates of switch rates are 1.2% with a range of (0.7%-1.2%) for males and 1.4% with a range of (1.1%-1.6%) for females. For ages 65-74 and 75+, the best estimates of switch rates are 0.6% with a range of (0.4%-1.9%) for males and 1.0% with a range of (0.3%-1.4%) for females.

**Table S2.1. The unweighted switch rates from current smokers to exclusive NVP users by age group, gender, and different NVP use definition using the prospective method definition 1***

|  |  | **Males** | | | | **Females** | | | |
| --- | --- | --- | --- | --- | --- | --- | --- | --- | --- |
| **Age** | **Transition** | **1+ days** | **5+ days** | **10+ days** | **20+ days** | **1+ days** | **5+ days** | **10+ days** | **20+ days** |
| **18+** | W1 ->W2 | 2.4% | 2.3% | 2.2% | 2.1% | 1.5% | 1.5% | 1.4% | 1.3% |
|  | W2->W3 | 2.5% | 2.3% | 2.2% | 2.0% | 1.5% | 1.3% | 1.2% | 1.1% |
|  | W3 ->W4 | 1.9% | 1.7% | 1.6% | 1.5% | 1.4% | 1.4% | 1.3% | 1.2% |
| **18-24** | W1 ->W2 | 3.3% | 3.1% | 2.9% | 2.7% | 1.6% | 1.6% | 1.3% | 1.2% |
|  | W2->W3 | 4.8% | 4.4% | 4.3% | 3.8% | 2.5% | 1.9% | 1.9% | 1.6% |
|  | W3 ->W4 | 4.3% | 3.9% | 3.8% | 3.8% | 2.1% | 1.8% | 1.6% | 1.4% |
| **25-34** | W1 ->W2 | 3.7% | 3.5% | 3.4% | 3.4% | 1.9% | 1.9% | 1.9% | 1.7% |
|  | W2->W3 | 2.8% | 2.5% | 2.3% | 2.2% | 1.6% | 1.3% | 1.2% | 1.2% |
|  | W3 ->W4 | 2.5% | 2.0% | 1.9% | 1.8% | 1.7% | 1.7% | 1.6% | 1.6% |
| **35-44** | W1 ->W2 | 2.0% | 2.0% | 2.0% | 2.0% | 1.5% | 1.4% | 1.4% | 1.2% |
|  | W2->W3 | 2.4% | 2.4% | 2.4% | 2.1% | 1.5% | 1.3% | 1.1% | 1.0% |
|  | W3 ->W4 | 1.8% | 1.6% | 1.5% | 1.3% | 1.1% | 0.9% | 0.9% | 0.9% |
| **45-54** | W1 ->W2 | 1.3% | 1.3% | 1.2% | 1.2% | 1.0% | 1.0% | 0.8% | 0.7% |
|  | W2->W3 | 1.0% | 0.8% | 0.8% | 0.7% | 0.9% | 0.8% | 0.8% | 0.8% |
|  | W3 ->W4 | 0.5% | 0.5% | 0.5% | 0.5% | 1.3% | 1.3% | 1.2% | 1.2% |
| **55-64** | W1 ->W2 | 1.2% | 1.2% | 1.0% | 1.0% | 2.0% | 1.9% | 1.7% | 1.5% |
|  | W2->W3 | 1.1% | 1.1% | 1.1% | 1.1% | 1.4% | 1.4% | 1.3% | 1.1% |
|  | W3 ->W4 | 0.2% | 0.2% | 0.2% | 0.2% | 1.1% | 1.1% | 1.1% | 1.0% |
| **65-74** | W1 ->W2 | 0.5% | 0.5% | 0.5% | 0.5% | 1.3% | 1.3% | 1.3% | 1.3% |
|  | W2->W3 | 1.4% | 1.4% | 1.4% | 0.9% | 0.9% | 0.9% | 0.5% | 0.5% |
|  | W3 ->W4 | 0.9% | 0.4% | 0.4% | 0.4% | 0.9% | 0.9% | 0.9% | 0.9% |
| **75+** | W1 ->W2 | 1.7% | 1.7% | 1.7% | 1.7% | 0.0% | 0.0% | 0.0% | 0.0% |
|  | W2->W3 | 0.0% | 0.0% | 0.0% | 0.0% | 1.8% | 1.8% | 1.8% | 1.8% |
|  | W3 ->W4 | 2.2% | 2.2% | 2.2% | 2.2% | 1.8% | 1.8% | 1.8% | 0.0% |

*Former smokers were defined as former smokers at wave t+1 and current smokers at wave t, t=1,2,3,4.

**Table S2.2. The relative difference in switch rates at distinct waves by age, gender, and NVP use definition using the prospective method definition 1***

| **Relative difference** %** | | **1+ days** | | **10+ days** | | **20+ days** | |
| --- | --- | --- | --- | --- | --- | --- | --- |
| **Age** | **Switch rates** | **M** | **F** | **M** | **F** | **M** | **F** |
| **18+** | W2 vs. W1 | 3.3% | 0.4% | 1.0% | -13.0% | -6.8% | -10.8% |
|  | W3 vs. W2 | -22.6% | -6.8% | -27.5% | 6.0% | -22.4% | 8.2% |
| **18-24** | W2 vs. W1 | 46.2% | 61.0% | 46.0% | 40.2% | 39.5% | 32.2% |
|  | W3 vs. W2 | -10.9% | -18.5% | -11.5% | -14.9% | -0.8% | -10.7% |
| **25-34** | W2 vs. W1 | -23.5% | -17.0% | -31.8% | -33.6% | -34.8% | -25.3% |
|  | W3 vs. W2 | -12.2% | 8.5% | -16.4% | 27.1% | -17.4% | 27.1% |
| **35-44** | W2 vs. W1 | 17.3% | 3.2% | 17.3% | -15.6% | 4.2% | -17.5% |
|  | W3 vs. W2 | -25.1% | -29.5% | -37.6% | -17.7% | -36.8% | -7.4% |
| **45-54** | W2 vs. W1 | -26.7% | -3.7% | -30.2% | -3.7% | -41.8% | 12.4% |
|  | W3 vs. W2 | -51.3% | 46.1% | -43.2% | 51.7% | -31.8% | 51.7% |
| **55-64** | W2 vs. W1 | -5.6% | -29.4% | 10.1% | -24.7% | 10.1% | -26.8% |
|  | W3 vs. W2 | -85.2% | -21.5% | -85.2% | -11.7% | -85.2% | -13.5% |
| **65-74** | W2 vs. W1 | 186.4% | -33.0% | 186.4% | -66.5% | 91.0% | -66.5% |
|  | W3 vs. W2 | -34.5% | 2.8% | -67.3% | 105.6% | -50.9% | 105.6% |

***** Former smokers were defined as former smokers at wave t+1 and current smokers at wave t, t=1,2,3,4.

**The negative (positive) value represents the reduction (increase) in wave t+1 vs wave t.

**Table S2.3. The unweighted switch rates from current smokers to exclusive NVP users by age group, gender, and different NVP use definition using the prospective method definition 2***

|  |  | **Males** | | | | **Females** | | | |
| --- | --- | --- | --- | --- | --- | --- | --- | --- | --- |
| **Age** | **Transition** | **1+ days** | **5+ days** | **10+ days** | **20+ days** | **1+ days** | **5+ days** | **10+ days** | **20+ days** |
| **18+** | W1 ->W2 | 2.4% | 2.3% | 2.2% | 2.2% | 1.5% | 1.5% | 1.4% | 1.3% |
|  | W2->W3 | 2.3% | 2.1% | 2.1% | 1.9% | 1.5% | 1.2% | 1.2% | 1.1% |
|  | W3 ->W4 | 1.7% | 1.4% | 1.4% | 1.4% | 1.3% | 1.3% | 1.2% | 1.1% |
| **18-24** | W1 ->W2 | 3.3% | 3.1% | 2.9% | 2.8% | 1.5% | 1.5% | 1.3% | 1.2% |
|  | W2->W3 | 4.8% | 4.3% | 4.2% | 3.7% | 2.6% | 1.9% | 1.9% | 1.6% |
|  | W3 ->W4 | 4.2% | 3.8% | 3.7% | 3.7% | 2.0% | 1.6% | 1.6% | 1.5% |
| **25-34** | W1 ->W2 | 3.6% | 3.4% | 3.3% | 3.3% | 1.9% | 1.9% | 1.9% | 1.7% |
|  | W2->W3 | 2.4% | 2.1% | 2.0% | 2.0% | 1.5% | 1.3% | 1.1% | 1.1% |
|  | W3 ->W4 | 2.0% | 1.5% | 1.4% | 1.4% | 1.6% | 1.6% | 1.5% | 1.5% |
| **35-44** | W1 ->W2 | 2.1% | 2.1% | 2.1% | 2.1% | 1.5% | 1.4% | 1.4% | 1.3% |
|  | W2->W3 | 2.3% | 2.3% | 2.3% | 2.0% | 1.3% | 1.2% | 1.0% | 0.9% |
|  | W3 ->W4 | 1.4% | 1.2% | 1.2% | 1.1% | 1.1% | 0.9% | 0.9% | 0.9% |
| **45-54** | W1 ->W2 | 1.3% | 1.3% | 1.2% | 1.2% | 1.0% | 1.0% | 0.8% | 0.7% |
|  | W2->W3 | 1.0% | 0.8% | 0.8% | 0.7% | 0.8% | 0.7% | 0.7% | 0.7% |
|  | W3 ->W4 | 0.5% | 0.5% | 0.5% | 0.5% | 1.2% | 1.2% | 1.1% | 1.1% |
| **55-64** | W1 ->W2 | 1.2% | 1.2% | 1.0% | 1.0% | 2.1% | 1.9% | 1.7% | 1.5% |
|  | W2->W3 | 1.1% | 1.1% | 1.1% | 1.1% | 1.4% | 1.4% | 1.3% | 1.1% |
|  | W3 ->W4 | 0.2% | 0.2% | 0.2% | 0.2% | 1.0% | 1.0% | 1.0% | 0.8% |
| **65-74** | W1 ->W2 | 0.5% | 0.5% | 0.5% | 0.5% | 1.4% | 1.4% | 1.4% | 1.4% |
|  | W2->W3 | 0.9% | 0.9% | 0.9% | 0.9% | 0.9% | 0.9% | 0.5% | 0.5% |
|  | W3 ->W4 | 0.5% | 0.0% | 0.0% | 0.0% | 0.5% | 0.5% | 0.5% | 0.5% |
| **75+** | W1 ->W2 | 2.0% | 2.0% | 2.0% | 2.0% | 0.0% | 0.0% | 0.0% | 0.0% |
|  | W2->W3 | 0.0% | 0.0% | 0.0% | 0.0% | 1.8% | 1.8% | 1.8% | 1.8% |
|  | W3 ->W4 | 2.2% | 2.2% | 2.2% | 2.2% | 1.8% | 1.8% | 1.8% | 0.0% |

*Using a stricter prospective definition, the selected former smokers were defined as former smokers who quit less than two years at wave t+1 and were current smokers at wave t, t=1,2,3,4.

**Table S2.4. The relative difference in switch rates at distinct waves by age, gender, and NVP use definition using the prospective method definition 2***

| **Relative difference** %** | | **1+ days** | | **10+ days** | | **20+ days** | |
| --- | --- | --- | --- | --- | --- | --- | --- |
| **Age group** | **Switch rates** | **M** | **F** | **M** | **F** | **M** | **F** |
| **18+** | W2 vs. W1 | -3.0% | -4.6% | -4.8% | -18.3% | -10.5% | -16.7% |
|  | W3 vs. W2 | -28.1% | -9.0% | -33.7% | 4.2% | -29.3% | 6.4% |
| **18-24** | W2 vs. W1 | 43.7% | 74.1% | 43.0% | 40.0% | 36.2% | 32.0% |
|  | W3 vs. W2 | -11.5% | -23.5% | -12.3% | -13.5% | -1.3% | -9.2% |
| **25-34** | W2 vs. W1 | -32.4% | -22.6% | -38.7% | -39.2% | -38.7% | -31.6% |
|  | W3 vs. W2 | -19.5% | 9.7% | -30.3% | 30.3% | -30.3% | 30.3% |
| **35-44** | W2 vs. W1 | 9.7% | -14.5% | 9.7% | -25.4% | -3.2% | -28.2% |
|  | W3 vs. W2 | -39.9% | -15.9% | -46.6% | -8.0% | -47.0% | 5.1% |
| **45-54** | W2 vs. W1 | -27.2% | -15.7% | -30.6% | -17.5% | -42.2% | -3.7% |
|  | W3 vs. W2 | -51.3% | 51.5% | -43.2% | 59.0% | -31.9% | 59.0% |
| **55-64** | W2 vs. W1 | -7.0% | -29.7% | 8.6% | -25.0% | 8.6% | -27.1% |
|  | W3 vs. W2 | -85.2% | -32.0% | -85.2% | -23.5% | -85.2% | -27.2% |
| **65-74** | W2 vs. W1 | 92.5% | -34.2% | 92.5% | -67.1% | 92.5% | -67.1% |
|  | W3 vs. W2 | -51.6% | -48.8% | -100.0% | 2.3% | -100.0% | 2.3% |

***** Using a stricter prospective definition, the selected former smokers were defined as former smokers who quit less than two years at wave t+1 and were current smokers at wave t, t=1,2,3,4.

**The negative (positive) value represents the reduction (increase) in wave t+1 vs wave t.

**Table S2.5. The unweighted switch rates from current smokers to exclusive NVP users by age group, gender, and different NVP use definition using the retrospective method**

|  |  | **Males** | | | | **Females** | | | |
| --- | --- | --- | --- | --- | --- | --- | --- | --- | --- |
| **Age** | **Wave** | **1+ days** | **5+ days** | **10+ days** | **20+ days** | **1+ days** | **5+ days** | **10+ days** | **20+ days** |
| **18+** | W1 | 1.3% | 1.3% | 1.3% | 1.2% | 1.6% | 1.6% | 1.6% | 1.5% |
|  | W2 | 2.8% | 2.6% | 2.4% | 2.2% | 1.8% | 1.7% | 1.6% | 1.4% |
|  | W3 | 3.1% | 2.7% | 2.5% | 2.2% | 1.9% | 1.6% | 1.4% | 1.2% |
|  | W4 | 3.3% | 2.8% | 2.7% | 2.5% | 2.0% | 1.8% | 1.6% | 1.5% |
| **18-24** | W1 | 1.2% | 1.2% | 1.1% | 1.0% | 1.3% | 1.2% | 1.1% | 1.0% |
|  | W2 | 4.4% | 4.0% | 3.5% | 3.0% | 2.3% | 1.7% | 1.3% | 1.1% |
|  | W3 | 5.8% | 4.7% | 4.3% | 3.6% | 3.5% | 2.3% | 2.0% | 1.5% |
|  | W4 | 7.5% | 6.1% | 5.8% | 5.2% | 3.6% | 3.0% | 2.7% | 2.3% |
| **25-34** | W1 | 1.9% | 1.8% | 1.8% | 1.7% | 1.7% | 1.7% | 1.6% | 1.6% |
|  | W2 | 3.9% | 3.6% | 3.4% | 3.1% | 1.9% | 1.9% | 1.9% | 1.6% |
|  | W3 | 3.2% | 2.8% | 2.6% | 2.4% | 1.7% | 1.5% | 1.2% | 1.2% |
|  | W4 | 3.2% | 3.0% | 2.8% | 2.8% | 2.2% | 2.1% | 1.7% | 1.7% |
| **35-44** | W1 | 1.2% | 1.1% | 1.1% | 1.1% | 1.6% | 1.6% | 1.6% | 1.5% |
|  | W2 | 1.8% | 1.8% | 1.8% | 1.8% | 2.1% | 2.0% | 2.0% | 1.9% |
|  | W3 | 2.5% | 2.3% | 2.2% | 2.0% | 2.0% | 1.9% | 1.8% | 1.7% |
|  | W4 | 2.2% | 2.0% | 1.8% | 1.5% | 1.3% | 1.1% | 1.1% | 1.1% |
| **45-54** | W1 | 1.2% | 1.1% | 1.1% | 1.0% | 1.8% | 1.8% | 1.8% | 1.6% |
|  | W2 | 1.3% | 1.3% | 1.2% | 1.2% | 1.3% | 1.3% | 1.2% | 1.1% |
|  | W3 | 1.6% | 1.4% | 1.4% | 1.2% | 0.7% | 0.7% | 0.7% | 0.7% |
|  | W4 | 1.4% | 1.4% | 1.4% | 1.4% | 1.6% | 1.6% | 1.4% | 1.4% |
| **55-64** | W1 | 1.0% | 1.0% | 1.0% | 0.9% | 1.7% | 1.7% | 1.7% | 1.7% |
|  | W2 | 1.3% | 1.3% | 1.2% | 1.2% | 1.7% | 1.6% | 1.4% | 1.3% |
|  | W3 | 1.7% | 1.6% | 1.6% | 1.6% | 2.0% | 1.7% | 1.6% | 1.5% |
|  | W4 | 0.2% | 0.2% | 0.2% | 0.2% | 0.9% | 0.9% | 0.9% | 0.8% |
| **65-74** | W1 | 1.0% | 1.0% | 1.0% | 1.0% | 1.3% | 1.3% | 1.3% | 1.3% |
|  | W2 | 0.7% | 0.7% | 0.7% | 0.7% | 1.4% | 1.4% | 1.4% | 1.4% |
|  | W3 | 0.7% | 0.7% | 0.7% | 0.7% | 0.7% | 0.7% | 0.4% | 0.4% |
| **65+*** | W4 | 0.5% | 0.3% | 0.3% | 0.3% | 0.5% | 0.5% | 0.5% | 0.3% |
| **75+** | W1 | 1.3% | 1.3% | 1.3% | 1.3% | 2.9% | 2.9% | 2.9% | 2.9% |
|  | W2 | 1.4% | 1.4% | 1.4% | 1.4% | 0.0% | 0.0% | 0.0% | 0.0% |
|  | W3 | 0.0% | 0.0% | 0.0% | 0.0% | 1.3% | 1.3% | 1.3% | 1.3% |

***** There are 7 age groups in waves 1, 2, and 3, i.e. age 18-24, 25-34, 35-44, 45-54, 55-64, 65-74, and 75 and above. There are 6 age groups in wave 4, i.e. age 18-24, 25-34, 35-44, 45-54, 55-64, 65 and above. Due to the small sample size of the age group 75 and above, we compare age group 65+ in wave 4 with age group 65-74 in other three waves in this paper.

**Table S2.6. The relative difference in switch rates at distinct waves by age, gender, and NVP use definition using the retrospective method**

| **Relative difference* %** | | **1+ days** | | **10+ days** | | **20+ days** | |
| --- | --- | --- | --- | --- | --- | --- | --- |
| **Age group** | **Switch rates** | **M** | **F** | **M** | **F** | **M** | **F** |
| **18+** | W2 vs. W1 | 107.2% | 14.1% | 89.9% | -0.1% | 86.4% | -8.1% |
|  | W3 vs. W2 | 12.8% | 4.9% | 5.4% | -9.5% | 1.5% | -9.3% |
|  | W4 vs. W3 | 5.6% | 1.3% | 7.6% | 11.8% | 12.1% | 18.9% |
| **18-24** | W2 vs. W1 | 267.6% | 77.7% | 213.9% | 18.3% | 198.8% | 2.5% |
|  | W3 vs. W2 | 32.0% | 50.0% | 21.7% | 50.6% | 19.9% | 36.9% |
|  | W4 vs. W3 | 29.6% | 3.1% | 35.2% | 31.6% | 45.2% | 59.7% |
| **25-34** | W2 vs. W1 | 101.3% | 13.4% | 84.8% | 18.8% | 79.6% | -1.0% |
|  | W3 vs. W2 | -18.8% | -10.5% | -21.8% | -34.9% | -23.3% | -26.7% |
|  | W4 vs. W3 | -0.7% | 29.5% | 6.6% | 40.3% | 16.0% | 49.6% |
| **35-44** | W2 vs. W1 | 55.1% | 29.7% | 68.0% | 23.2% | 68.0% | 24.0% |
|  | W3 vs. W2 | 37.5% | -3.3% | 19.6% | -8.9% | 7.6% | -9.5% |
|  | W4 vs. W3 | -13.0% | -34.1% | -18.1% | -36.8% | -24.2% | -32.9% |
| **45-54** | W2 vs. W1 | 9.9% | -29.0% | 9.0% | -34.9% | 19.9% | -34.2% |
|  | W3 vs. W2 | 26.7% | -43.8% | 17.9% | -38.7% | 7.2% | -32.6% |
|  | W4 vs. W3 | -12.8% | 117.0% | 3.1% | 89.9% | 13.4% | 89.9% |
| **55-64** | W2 vs. W1 | 33.2% | 1.3% | 19.9% | -15.6% | 37.0% | -24.0% |
|  | W3 vs. W2 | 29.5% | 17.7% | 32.8% | 13.0% | 32.8% | 15.1% |
|  | W4 vs. W3 | -85.9% | -52.8% | -84.7% | -41.0% | -84.7% | -43.7% |
| **65-74** | W2 vs. W1 | -28.8% | 8.2% | -28.8% | 8.2% | -28.8% | 8.2% |
|  | W3 vs. W2 | -4.8% | -48.7% | -4.8% | -74.4% | -4.8% | -74.4% |
|  | W4 vs. W3 | -27.3% | -30.8% | -63.6% | 38.4% | -63.6% | -30.8% |

*The negative (positive) value represents the reduction (increase) in wave t+1 vs wave t.

**Table S2.7. Unweighted average switch rates for all waves 1-4 by gender, age, and NVP use definition**

|  |  | **Males** | | | | **Females** | | | |
| --- | --- | --- | --- | --- | --- | --- | --- | --- | --- |
| **Age** | **Definition*** | **1+ days** | **5+ days** | **10+ days** | **20+ days** | **1+ days** | **5+ days** | **10+ days** | **20+ days** |
| **18+** | Prospective Def 1 | 2.3% | 2.1% | 2.0% | 1.9% | 1.5% | 1.4% | 1.3% | 1.2% |
|  | Prospective Def 2 | 2.2% | 2.0% | 1.9% | 1.8% | 1.4% | 1.3% | 1.3% | 1.2% |
|  | Retrospective | 2.6% | 2.3% | 2.2% | 2.0% | 1.8% | 1.6% | 1.5% | 1.4% |
| **18-24** | Prospective Def 1 | 4.1% | 3.7% | 3.6% | 3.4% | 2.0% | 1.7% | 1.6% | 1.4% |
|  | Prospective Def 2 | 4.1% | 3.7% | 3.6% | 3.3% | 2.0% | 1.7% | 1.6% | 1.4% |
|  | Retrospective | 4.7% | 4.0% | 3.7% | 3.2% | 2.7% | 2.1% | 1.8% | 1.5% |
| **25-34** | Prospective Def 1 | 3.0% | 2.7% | 2.6% | 2.5% | 1.7% | 1.6% | 1.6% | 1.5% |
|  | Prospective Def 2 | 2.7% | 2.4% | 2.3% | 2.3% | 1.6% | 1.6% | 1.5% | 1.4% |
|  | Retrospective | 3.0% | 2.8% | 2.6% | 2.5% | 1.9% | 1.8% | 1.6% | 1.5% |
| **35-44** | Prospective Def 1 | 2.1% | 2.0% | 2.0% | 1.9% | 1.4% | 1.2% | 1.2% | 1.1% |
|  | Prospective Def 2 | 1.9% | 1.9% | 1.9% | 1.7% | 1.3% | 1.2% | 1.1% | 1.0% |
|  | Retrospective | 1.9% | 1.8% | 1.7% | 1.6% | 1.7% | 1.6% | 1.6% | 1.5% |
| **45-54** | Prospective Def 1 | 1.0% | 0.9% | 0.9% | 0.8% | 1.1% | 1.0% | 0.9% | 0.9% |
|  | Prospective Def 2 | 1.0% | 0.9% | 0.9% | 0.8% | 1.0% | 1.0% | 0.9% | 0.8% |
|  | Retrospective | 1.4% | 1.3% | 1.2% | 1.2% | 1.4% | 1.4% | 1.3% | 1.2% |
| **55-64** | Prospective Def 1 | 0.8% | 0.8% | 0.8% | 0.8% | 1.5% | 1.5% | 1.4% | 1.2% |
|  | Prospective Def 2 | 0.8% | 0.8% | 0.8% | 0.8% | 1.5% | 1.4% | 1.3% | 1.2% |
|  | Retrospective | 1.0% | 1.0% | 1.0% | 0.9% | 1.6% | 1.5% | 1.4% | 1.3% |
| **65-74** | Prospective Def 1 | 0.9% | 0.8% | 0.8% | 0.6% | 1.1% | 1.1% | 0.9% | 0.9% |
|  | Prospective Def 2 | 0.6% | 0.5% | 0.5% | 0.5% | 0.9% | 0.9% | 0.8% | 0.8% |
|  | Retrospective | 0.7% | 0.6% | 0.6% | 0.6% | 1.0% | 1.0% | 0.9% | 0.8% |
| **75+** | Prospective Def 1 | 1.3% | 1.3% | 1.3% | 1.3% | 1.3% | 1.3% | 1.3% | 0.6% |
|  | Prospective Def 2 | 1.4% | 1.4% | 1.4% | 1.4% | 1.3% | 1.3% | 1.3% | 0.6% |
|  | Retrospective | 0.9% | 0.9% | 0.9% | 0.9% | 1.4% | 1.4% | 1.4% | 1.4% |

*** F**ormer smokers were defined as former smokers at wave t+1 and current smokers at wave t, t=1,2,3,4. Using Prospective Def 2 (a strict definition), the selected former smokers were defined as former smokers who quit less than two years at wave t+1 and were current smokers at wave t.

**Table S2.8. Weighted average transition rates for all waves 1-4 by gender, age, and NVP use definition**

|  |  | **Males** | | | | **Females** | | | |
| --- | --- | --- | --- | --- | --- | --- | --- | --- | --- |
| **Age** | **Definition*** | **1+ days** | **5+ days** | **10+ days** | **20+ days** | **1+ days** | **5+ days** | **10+ days** | **20+ days** |
| **18+** | Prospective Def 1 | 2.1% | 1.9% | 1.9% | 1.8% | 1.6% | 1.5% | 1.3% | 1.2% |
|  | Prospective Def 2 | 2.0% | 1.8% | 1.8% | 1.7% | 1.5% | 1.4% | 1.3% | 1.2% |
|  | Retrospective | 2.4% | 2.1% | 2.0% | 1.9% | 1.8% | 1.6% | 1.5% | 1.4% |
| **18-24** | Prospective Def 1 | 4.3% | 3.9% | 3.8% | 3.6% | 2.5% | 2.1% | 1.9% | 1.7% |
|  | Prospective Def 2 | 4.3% | 3.9% | 3.8% | 3.5% | 2.5% | 2.0% | 2.0% | 1.7% |
|  | Retrospective | 4.8% | 4.1% | 3.8% | 3.4% | 3.1% | 2.2% | 2.0% | 1.7% |
| **25-34** | Prospective Def 1 | 2.8% | 2.6% | 2.5% | 2.4% | 2.1% | 2.0% | 1.8% | 1.6% |
|  | Prospective Def 2 | 2.6% | 2.3% | 2.2% | 2.2% | 2.0% | 2.0% | 1.8% | 1.6% |
|  | Retrospective | 3.1% | 2.9% | 2.8% | 2.6% | 2.2% | 2.0% | 1.8% | 1.6% |
| **35-44** | Prospective Def 1 | 2.2% | 1.8% | 1.8% | 1.7% | 1.4% | 1.2% | 1.1% | 1.1% |
|  | Prospective Def 2 | 2.1% | 1.7% | 1.7% | 1.6% | 1.3% | 1.1% | 1.1% | 1.0% |
|  | Retrospective | 2.0% | 1.6% | 1.6% | 1.4% | 1.8% | 1.6% | 1.6% | 1.5% |
| **45-54** | Prospective Def 1 | 1.0% | 1.0% | 0.9% | 0.9% | 1.0% | 1.0% | 0.9% | 0.9% |
|  | Prospective Def 2 | 1.0% | 1.0% | 0.9% | 0.9% | 0.9% | 0.9% | 0.8% | 0.8% |
|  | Retrospective | 1.3% | 1.2% | 1.2% | 1.2% | 1.2% | 1.2% | 1.1% | 1.0% |
| **55-64** | Prospective Def 1 | 0.8% | 0.8% | 0.7% | 0.7% | 1.4% | 1.4% | 1.3% | 1.2% |
|  | Prospective Def 2 | 0.8% | 0.8% | 0.8% | 0.8% | 1.4% | 1.3% | 1.3% | 1.1% |
|  | Retrospective | 1.1% | 1.0% | 1.0% | 1.0% | 1.6% | 1.5% | 1.5% | 1.4% |
| **65-74** | Prospective Def 1 | 0.9% | 0.7% | 0.7% | 0.5% | 1.4% | 1.4% | 1.1% | 1.1% |
|  | Prospective Def 2 | 0.6% | 0.4% | 0.4% | 0.4% | 1.0% | 1.0% | 0.7% | 0.7% |
|  | Retrospective | 0.7% | 0.5% | 0.5% | 0.5% | 1.0% | 1.0% | 0.9% | 0.8% |
| **75+** | Prospective Def 1 | 1.7% | 1.7% | 1.7% | 1.7% | 0.9% | 0.9% | 0.9% | 0.3% |
|  | Prospective Def 2 | 1.9% | 1.9% | 1.9% | 1.9% | 0.9% | 0.9% | 0.9% | 0.3% |
|  | Retrospective | 1.3% | 1.3% | 1.3% | 1.3% | 1.4% | 1.4% | 1.4% | 1.4% |

*** F**ormer smokers were defined as former smokers at wave t+1 and current smokers at wave t, t=1,2,3,4. Using Prospective Def 2 (a strict definition), the selected former smokers were defined as former smokers who quit less than two years at wave t+1 and were current smokers at wave t.

**Table S2.9. Our best estimates for switch rates by age and gender from PATH waves 1 to 4**

|  | **Male** | | **Female** | |
| --- | --- | --- | --- | --- |
| **Ages** | **Rate** | **Overall range** | **Rate** | **Overall range** |
| **18-24** | 4.0% | 3.4% - 4.8% | 2.5% | 1.7% - 3.1% |
| **25-34** | 2.5% | 2.2% - 3.1% | 2.0% | 1.6% - 2.2% |
| **35-45** | 2.5% | 1.4% - 2.5% | 1.6% | 1.0% - 1.8% |
| **45-54** | 1.3% | 0.9% - 1.3% | 1.4% | 0.8% - 1.4% |
| **55-64** | 1.2% | 0.7% - 1.2% | 1.4% | 1.1% - 1.6% |
| **65+** | 0.6% | 0.4% - 1.9% | 1.0% | 0.3% - 1.4% |

Notes: All switch rates and ranges were estimated by using the weighted rates from PATH, and the age group 65-74 and 75+ in waves 1,2, and 3 were combined.

**Supplement 3. Further validation of the model**

In the paper, we validated the SAVM against data from the NHIS. In this supplement, we validate smoking prevalence against the TUS-CPS (Tobacco Use Supplement to the Current Population Survey) and NSDUH (National Survey on Drug Use and Health) by age and gender over the years 2013-2018. Due to the unavailability of data in the TUS-CPS for the year 2013, we applied a weighted averaged of the prevalence rate from TUS-CPS in 2010/11 (60% weight) and 2014/15 (40% weight) to approximate the rate in 2013. For the TUS-CPS, data is available by age and gender, while for the NSDUH, data is available by gender or by age. Consequently, we validated the smoking rate in the model by age and gender together against TUS-CPS and by age and by gender separately against NSDUH. We also validate the NVP prevalence against data from the TUS-CPS, where the NVP prevalence is measured by daily or someday use.

**Validation of Smoking Prevalence**

Using the initial (best estimate) input parameter estimates, the SAVM predictions are validated against smoking prevalence from NSDUH and TUS-CPS data for 2013-2018 as shown in Table S.3.1.

For ages 18 and above, SAVM projects that male (female) smoking prevalence fell from 21.4% (15.9%) in 2013 to 16.7% (12.6%) in 2018, while NSDUH shows a decline from 23.8% (18.4%) in 2013 to 19.4% (15.3%) in 2018 and TUS-CPS declines from 16.6% (13.1%) in 2013 to 14.3% (11.6%) in 2018. NSDUH and TUS-CPS show 18.3% (17.3%) and 13.7% (11.3%) relative reductions between 2013 and 2018 compared to a 22.2% (20.7%) relative reduction in SAVM.

The smoking prevalence for males ages 18 and above is shown in Figure S.3.1.a., with scaled male SAVM prevalence rates calculated as the male SAVM prevalence rates in 2013-2018 multiplied by the ratio of the survey to SAVM levels in 2013, e.g. NSDUH 23.8%/ SAVM 21.4% = 1.11 for NSDUH). Figure S.3.1.b. shows female prevalence rates, also scaled by the ratio of survey estimate to SAVM in 2013.

By age group, SAVM projects that male (female) prevalence for ages 18-24 fell by 33.0% (33.7%) in relative terms between 2013 and 2018 compared to 29.3% (28.3%) from TUS-CPS over the same period. For ages 25-44, SAVM projects a relative decline of 21.0% (17.6%) compared to 14.7% (12.9%) from TUS-CPS. For ages 45-64, SAVM projects a relative decline of 19.9% (18.1%) compared to 6.6% (2.8%) from TUS-CPS. For ages 65 and above, SAVM projects a relative decline of 16.6% (20.3%) compared to a 0.3% increase (2.8% decrease) from TUS-CPS.

Combining males and females, SAVM projects that the combined prevalence for age 18-25 fell by 33.3% in relative terms in 2013-2018 compared to 39.6% from NSDUH over the same period. For age 26-34, SAVM projects a relative decline of 26.5% compared to 24.5% from NSDUH. For age 35-49, SAVM projects a relative decline of 11.7% compared to 10.4% from NSDUH. For age 50 and above, SAVM projects a relative decline of 20.6% compared to 8.0% from NSDUH.

While SAVM estimates for 2018 are often outside the confidence intervals of the survey estimate, upon scaling all years by the initial year (2013) from SAVM relative to the initial year in the survey estimates for comparability, the SAVM 2018 estimates are generally within the confidence intervals, with the exceptions of the age 45-64 and 65+ for both genders from the TUS-CPS and age 50+ for both genders from NSDUH in recent years (2016-2018).

**Validation of NVP Prevalence**

The SAVM predictions for exclusive NVP use are validated against the overall NVP prevalence for both genders combined from TUS-CPS data for 2018 as shown in Table S.3.2, where NVP use is measures as every day or someday use.

For ages 18 and above, SAVM projects 2018 NVP prevalence of 2.4% for both genders combined compared to 1.4% (95% CI: 1.3%-1.5%) from TUS-CPS. By age group for both genders combined, the SAVM projection against TUS-CPS comparisons (with confidence intervals) for 2018 are 6.2% vs 4.0% (95% CI:3.5%-4.5%) for ages 18-24, 3.3% vs 2.1% (95% CI:1.9%-2.4%) for ages 25-34, 1.2% vs 1.1% (95% CI:1.0%-1.2%) for ages 35-54, and 0.5% vs 0.5% (95% CI:0.5%-0.6%) for ages 55 and above. SAVM predictions are above the 95% confidence intervals estimated from TUS-CPS for overall and by age groups, except for age 35-54 and 55 and above that fell within the intervals.

**Table S.3.1.** **Smoking prevalence (%), validation of US SAVM against the TUS-CPS, and NSDUH, by age and/or gender, 2013-2018**

| **Age** | **Source** | **2013** | **2014** | **2015** | **2016** | **2017** | **2018** | **Relative difference 2013-18** |  |
| --- | --- | --- | --- | --- | --- | --- | --- | --- | --- |
| **US Males** | | | | | | | | | |
| **18+** | **SAVM** | 21.4 | 20.4 | 19.4 | 18.5 | 17.5 | 16.7 | -22.20% |  |
|  | **NSDUH** | 23.8 | 23.6 | 22 | 21.1 | 20.7 | 19.4 | -18.30% |  |
|  | **95% CI** | 22.7-24.9 | 22.9-24.3 | 21.2-22.8 | 20.3-21.8 | 19.9-21.4 | 18.6-20.3 |  |  |
|  | **CPS-TUS** | 16.6 | - | 15.6 | - | - | 14.3 | -13.70% |  |
|  | **95% CI** | - | - | 15.1-16.1 | - | - | 13.8-14.8 |  |  |
| **18-24** | **SAVM** | 19.9 | 18.2 | 16.6 | 15.3 | 14.2 | 13.3 | -33.0% |  |
|  | **TUS-CPS** | 16.9 | - | 13.3 | - | - | 12.0 | -29.3% |  |
|  | **95% CI** | - | - | 12.7-13.9 | - | - | 10.2-13.9 |  |  |
| **25-44** | **SAVM** | 27.3 | 26.3 | 25.2 | 24 | 22.8 | 21.6 | -21.0% |  |
|  | **TUS-CPS** | 19.1 | - | 18.3 | - | - | 16.0 | -14.7% |  |
|  | **95% CI** | - | - | 17.9-18.7 | - | - | 15.5-17.2 |  |  |
| **45-64** | **SAVM** | 20.4 | 19.5 | 18.6 | 17.8 | 17.1 | 16.3 | -19.9% |  |
|  | **TUS-CPS** | 18.2 | - | 17.5 | - | - | 17.0 | -6.6% |  |
|  | **95% CI** | - | - | 17.1-17.9 | - | - | 16.2-17.9 |  |  |
| **65+** | **SAVM** | 12.2 | 11.9 | 11.5 | 11 | 10.6 | 10.2 | -16.6% |  |
|  | **TUS-CPS** | 8.6 | - | 8.8 | - | - | 8.7 | 0.3% |  |
|  | **95% CI** | - | - | 8.4-9.1 | - | - | 7.9-9.4 |  |  |
| **US Females** | | | | | | | | | |
| **18+** | **SAVM** | 15.9 | 15.2 | 14.5 | 13.8 | 13.2 | 12.6 | -20.7% |  |
|  | **NSDUH** | 18.4 | 18.4 | 16.7 | 17.2 | 15.1 | 15.3 | -17.3% |  |
|  | **95%CI** | 17.6-19.2 | 17.6-19.1 | 16.0-17.4 | 16.4-18.0 | 14.4-15.7 | 14.6-15.9 |  |  |
|  | **TUS-CPS** | 13.1 | - | 12.1 | - | - | 11.6 | -11.3% |  |
|  | **95%CI** | - | - | 11.7-12.5 | - | - | 11.2-12.0 |  |  |
| **18-24** | **SAVM** | 15 | 13.7 | 12.5 | 11.5 | 10.6 | 9.9 | -33.7% |  |
|  | **TUS-CPS** | 12.5 | - | 9.2 | - | - | 9.0 | -28.3% |  |
|  | **95% CI** | - | - | 8.7-9.7 | - | - | 7.6-10.6 |  |  |
| **25-44** | **SAVM** | 20.6 | 19.9 | 19.2 | 18.5 | 17.7 | 16.9 | -17.6% |  |
|  | **TUS-CPS** | 14.9 | - | 13.8 | - | - | 13.0 | -12.9% |  |
|  | **95% CI** | - | - | 13.5-14.1 | - | - | 12.3-13.7 |  |  |
| **45-64** | **SAVM** | 16.2 | 15.6 | 14.9 | 14.4 | 13.8 | 13.3 | -18.1% |  |
|  | **TUS-CPS** | 15.4 | - | 14.7 | - | - | 15.0 | -2.8% |  |
|  | **95% CI** | - | - | 14.3-15.0 | - | - | 14.2-15.8 |  |  |
| **65+** | **SAVM** | 8.2 | 7.8 | 7.4 | 7.1 | 6.8 | 6.5 | -20.3% |  |
|  | **TUS-CPS** | 6.8 | - | 6.8 | - | - | 6.6 | -2.8% |  |
|  | **95% CI** | - | - | 6.5-7.1 | - | - | 6.1-7.2 |  |  |

| **US Males and Females Combined** | | | | | | | | |
| --- | --- | --- | --- | --- | --- | --- | --- | --- |
| **18-25** | **SAVM** | 18.3 | 16.7 | 15.3 | 14.1 | 13.1 | 12.2 | -33.3% |
|  | **NSDUH** | 23.6 | 22.47 | 20.5 | 17.71 | 16.88 | 14.25 | -39.6% |
|  | **95% CI** | 22.7-24.5 | 21.2-23.7 | 19.7-21.4 | 16.8-18.6 | 16.1-17.7 | 13.5-15.0 |  |
| **26-34** | **SAVM** | 26.4 | 25.2 | 23.8 | 22.4 | 20.9 | 19.4 | -26.5% |
|  | **NSDUH** | 31.0 | 27.03 | 26.8 | 24.86 | 24.06 | 23.4 | -24.5% |
|  | **95% CI** | 29.5-32.5 | 25.8-28.3 | 25.6-27.9 | 23.7-26.1 | 23.0-25.1 | 22.2-24.6 |  |
| **35-49** | **SAVM** | 20.7 | 20.2 | 19.7 | 19.2 | 18.8 | 18.3 | -11.7% |
|  | **NSDUH** | 23.2 | 23.9 | 21.8 | 21.71 | 21.21 | 20.83 | -10.4% |
|  | **95% CI** | 22.1-24.4 | 22.8-24.9 | 20.8-22.9 | 20.6-22.8 | 20.3-22.2 | 19.8-21.8 |  |
| **50+** | **SAVM** | 14.7 | 14.0 | 13.4 | 12.8 | 12.2 | 11.7 | -20.6% |
|  | **NSDUH** | 15.3 | 16.5 | 14.8 | 16.01 | 13.92 | 14.07 | -8.0% |
|  | **95% CI** | 14.1-16.5 | 15.7-17.4 | 13.9-15.7 | 15.0-17.0 | 13.1-14.7 | 13.2-14.9 |  |

Notes: Current smokers in the TUS-CPS (Tobacco Use Supplement to the Current Population Survey) and NSDUH (National Survey on Drug Use and Health) are those who have smoked at least 100 cigarettes lifetime, and smoked at least 1 days in the last 30 days

**Table S.3.2. Nicotine vaping product prevalence (%), validation for US SAVM against TUS-CPS for males and females combined by age in year 2018**

| **TUS-CPS - US males and females combined** | | | | | |
| --- | --- | --- | --- | --- | --- |
| **Ages** | **18+** | **18-24** | **25-34** | **35-54** | **55+** |
| **SAVM** | 2.4 | 6.2 | 3.3 | 1.2 | 0.5 |
| **TUS-CPS** | 1.4 | 4.0 | 2.1 | 1.1 | 0.5 |
| **95% CI** | 1.3-1.5 | 3.5-4.5 | 1.9-2.4 | 1.0-1.2 | 0.5-0.6 |

Notes: CI= confidence interval. NVP users from TUS-CPS (Tobacco Use Supplement to the Current Population Survey) are those who use NVPs every day or somedays.

**Figure S.3.1.a.** **Male smoking prevalence (ages 18 and above), Original and Scaled SAVM, NSDUH, and TUS-CPS estimates, 2013- 2018**

**Figure S.3.1.b.** **Female smoking prevalence (ages 18 and above), Original and Scaled SAVM, NSDUH, and TUS-CPS estimates, 2013- 2018**
